# Web-Based Nomograms for Overall Survival and Cancer-Specific Survival of Bladder Cancer Patients with Bone Metastasis: A Retrospective Cohort Study from SEER Database

**DOI:** 10.3390/jcm12020726

**Published:** 2023-01-16

**Authors:** Sheng Yang, Hongmin Zhou, Chaobo Feng, Ningze Xu, Yunshan Fan, Zhi Zhou, Yunfei Xu, Guoxin Fan, Xiang Liao, Shisheng He

**Affiliations:** 1 Department of Orthopedics, Shanghai Tenth People’s Hospital, Tongji University School of Medicine, Shanghai 200072, China; 2 Spinal Pain Research Institute, Tongji University School of Medicine, Shanghai 200072, China; 3 Department of Urology, Shanghai Tenth People’s Hospital, Tongji University School of Medicine, Shanghai 200072, China; 4 Department of Obstetrics and Gynecology, Shanghai Tenth People’s Hospital, Tongji University School of Medicine, Shanghai 200072, China; 5 National Key Clinical Pain Medicine of China, Huazhong University of Science and Technology Union Shenzhen Hospital, Shenzhen 518052, China; 6 Guangdong Key Laboratory for Biomedical Measurements and Ultrasound Imaging, School of Biomedical Engineering, Shenzhen University Health Science Center, Shenzhen 518060, China; 7 Department of Spine Surgery, Third Affiliated Hospital, Sun Yat-sen University, Guangzhou 510630, China

**Keywords:** SEER, prognostic factor, bladder cancer, bone metastasis, survival prediction, nomogram

## Abstract

Background: Our study aimed to explore the prognostic factors of bladder cancer with bone metastasis (BCBM) and develop prediction models to predict the overall survival (OS) and cancer-specific survival (CSS) of BCBM patients. Methods: A total of 1438 patients with BCBM were obtained from the SEER database. Patients from 2010 to 2016 were randomly divided into training and validation datasets (7:3), while patients from 2017 were divided for external testing. Nomograms were established using prognostic factors identified through Cox regression analyses and validated internally and externally. The concordance index (C-index), calibration plots, and time-dependent receiver operating characteristic (ROC) curves were used to evaluate the discrimination and calibration of nomogram models, while decision curve analyses (DCA) and Kaplan-Meier (KM) curves were used to estimate the clinical applicability. Results: Marital status, tumor metastasis (brain, liver, and lung), primary site surgery, and chemotherapy were indicated as independent prognostic factors for OS and CSS. Calibration plots and the overall C-index showed a novel agreement between the observed and predicted outcomes. Nomograms revealed significant advantages in OS and CSS predictions. AUCs for internal and external validation were listed as follows: for OS, 3-month AUCs were 0.853 and 0.849; 6-month AUCs were 0.873 and 0.832; 12-month AUCs were 0.825 and 0.805; for CSS, 3-month AUCs were 0.849 and 0.847; 6-month AUCs were 0.870 and 0.824; 12-month AUCs were 0.815 and 0.797, respectively. DCA curves demonstrated good clinical benefit, and KM curves showed distinct stratification performance. Conclusion: The nomograms as web-based tools were proved to be accurate, efficient, and clinically beneficial, which might help in patient management and clinical decision-making for BCBM patients.

## 1. Introduction

Bladder cancer (BCa) is the second most common genitourinary cancer and the fourth most common male cancer [1]. According to statistics, in 2020, there were 573,278 new BCa cases worldwide and about 212,536 deaths [2]. Approximately 10–15% of BCa patients have already developed metastases at the time of initial diagnosis [3]. Metastatic bladder cancer (mBC) has a poor prognosis, with a 5-year survival rate of only 10% [4]. Bone is the most common site of organ metastasis in bladder cancer [5,6,7]. Bone metastasis can cause skeletal related events (SREs) and reduce the survival rates of cancer patients [8]. Therefore, it is of great importance to evaluate the survival and prognosis of bladder cancer with bone metastasis (BCBM).

The TNM staging system is widely used in the prognostic assessment of cancer patients and is often used by clinicians to develop treatment plans [9]. Studies have shown that age, gender, race, and treatment modality can also affect the prognosis of patients with BCa [10,11]. The TNM staging system does not adequately cover the biological characteristics of the cancer and the information on the treatment of the tumor, and the prognosis of BCa patients varies depending on the metastatic organs [12]. Therefore, the prediction accuracy of the TNM staging system may be reduced for patients with distant metastases.

As simple, user-friendly statistical prediction models, nomograms have already been widely utilized for prognosis prediction of cancer patients in recent years [13,14,15,16]. By integrating important demographic and clinicopathological variables, nomograms can accurately predict individual patient survival. However, to date, no prognostic model has been developed for patients with BCBM. In this study, we obtained the clinical data of patients with BCBM from the surveillance, epidemiology, and end results (SEER) database. We used COX regression analysis to identify overall survival (OS)-related prognostic factors and cancer-specific survival (CSS)-related prognostic factors. Based on the above results, we further developed and validated two web-based nomograms for the prognostic prediction of patients with BCBM.

## 2. Methods

### 2.1. Sources of Databases

All registered cases of BCBM diagnosed between 2010 and 2017 were obtained from the SEER database. SEER contains clinical information on patients from 18 cancer registries and covers approximately 28% of the whole United States population. Collection of SEER includes patients’ demographic characteristics, tumor histological characteristics, treatment, and follow-up information. Ethical approval for this study was not required because the SEER database contained no private patient information.

### 2.2. Patient and Public Involvement

Patients and/or the public were not involved in the design, conduct, reporting, or dissemination plans of this research.

### 2.3. Inclusion and Exclusion Criteria

Demographic and clinical data available in the SEER database were extracted, including age, gender, race (black, white, other race), marital status (married, unmarried), histological type, tumor grade, T stage, N stage, metastatic sites (brain, liver, and lung), primary site surgery, radiotherapy, and chemotherapy.

**Figure 1:**
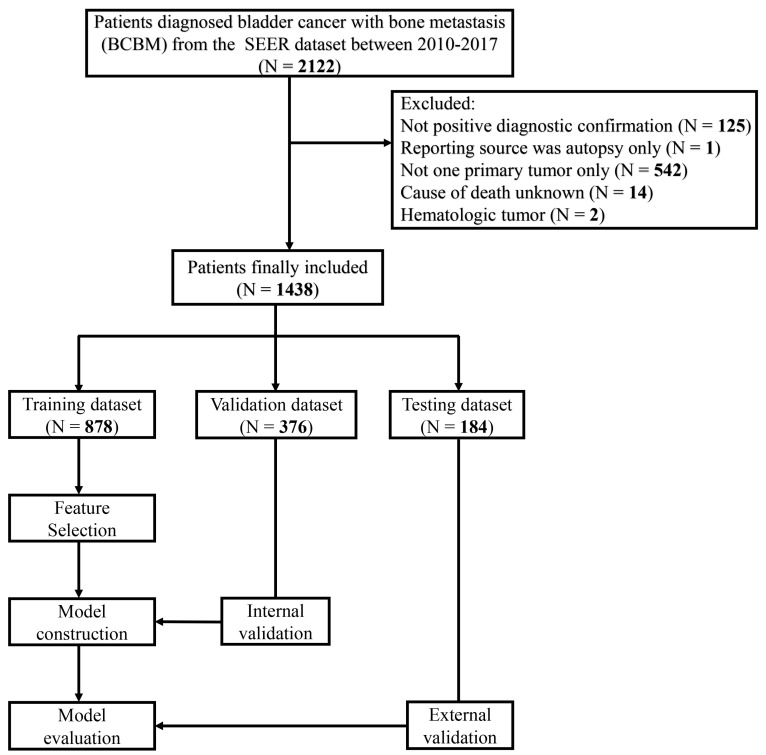
Workflow of the patient selection and model development.

The inclusion criteria were as follows: (1) Bladder site record (C67.0-C67.9) according to the Third Edition of International Classification of Diseases for Oncology (ICD-O-3); (2) Diagnosed with bone metastasis. The exclusion criteria were as follows: (1) Patients without positive diagnostic confirmation; (2) Patients diagnosed with autopsy only; (3) Patients with more than one primary tumor (for example, prostate cancer, et al.); (4) Unknown cause of death; (5) Hematologic tumor. The flowchart of this study is shown in Figure 1.

### 2.4. Feature Selection

We randomly allocated patients from 2010 to 2016 to a training dataset of 70% patients and an internal validation dataset of 30% patients. Patients in 2017 were selected for external testing, namely the testing dataset. Univariable and stepwise, backward, multivariable Cox regression analyses were conducted on the training dataset to identify the independent prognostic variables. Each variable’s contribution to the final regression model was measured as the partial chi-square statistic minus the variable degrees of freedom (χ^2^−df).

### 2.5. Construction and Validation of The nomograms

Among the important variables selected, visual nomogram models for OS and CSS of patients with BCBM at 3-months, 6-months, and 12-months were constructed on the training dataset. We obtained a specific score for each variable in the constructed nomograms and quantified patients’ OS and CSS rates as risk scores on a number line. Then, X-tile software (version 3.5; developed by Yale University, New Haven, CT, USA.) was utilized to determine the best cut-off values of nomogram total risk scores for OS and CSS, and patients were categorized into high-risk and low-risk groups according to the cut-off values. Kaplan–Meier (KM) curves and risk scatterplots were utilized to evaluate the stratification performance of the risk score and risk distribution in patients with BCBM.

For the predictive reliability and accuracy of the nomograms, internal validation and external testing were conducted on the validation datasets and the testing datasets, respectively. Calibration plots were used to validate the nomograms’ calibration. In addition, the area under the curves (AUC) of the receiver operating characteristic (ROC) curve and the overall Harrel’s concordance index (C-index) were constructed to reflect the discriminability of the nomograms. A decision curve analysis (DCA) was conducted to evaluate the clinical net benefit of the nomogram. In comparison, the predictive performance of the top three important indicators was evaluated separately in the same way as the nomogram. The prediction errors of different models were measured using the integrated Brier score (IBS), also known as the prediction error rate. A lower IBS indicates a higher prediction performance. An IBS value of 0.5 or lower means that the model’s predictive ability is better than a chance.

### 2.6. Statistical Analysis

All statistical analyses were conducted on R software (version 3.6.1; R Foundation for Statistical Computing, Vienna, Austria; https://www.r-project.org, accessed on 1 July 2022.). The R packages “mice” and “VIM” were used for imputation of the missing values, while “rms”, “survival”, “DynNom”, “pec”, and “ggDCA” were used for constructing and validating the nomograms. Multiple imputations (100 imputations) were used to estimate the missing values. All evaluation parameters were calculated with 1000 bootstrap resamples. Categorical variables were reported as counts (percentage), and continuous variables were summarized as means (standard deviation) and compared using the Chi-square test and one-way ANOVA, respectively. Two-sided *p*-value < 0.05 indicated statistical significance.

## 3. Results

### 3.1. Patient Characteristics

A total of 1438 patients with BCBM were extracted for analysis, including 878 patients in the training dataset, 376 in the validation dataset, and 184 in the testing dataset. As shown in Table 1, the mean survival time was 7.89 ± 10.85, 8.29 ± 11.11, and 7.76 ± 11.98 months in the whole dataset, the training cohort, and the validation cohort, respectively. Until the last follow-up, 77 (5.4%) patients were alive for OS and CSS (34 in the training dataset, 9 in the internal validation dataset, and 34 in the testing dataset), in addition to which 1274 (88.6%) patients died of cancer and 87 (6.1%) patients died of other causes. Cumulative incidence curves of cancer and noncancer deaths in the whole dataset were demonstrated in Supplementary Figure S1. The distribution of age is shown in Supplementary Figure S2. Among all patients, 715 (49.7%) were married and 723 (50.3%) were unmarried; 48 (3.3%) were diagnosed with brain metastasis, 348 (24.2%) with liver metastasis, and 405 (28.2%) with lung metastasis; 58 (4.0%) were conducted with a complete cystectomy, 984 (68.4%) with a non-complete cystectomy, and 682 (47.4%) patients received chemotherapy. Demographic and clinical data categorized by different survival outcomes were displayed in Supplementary Table S1. Imputation of baseline characteristics brought no significant difference to the whole dataset (*p* > 0.05), as shown in Supplementary Table S2. Missing data were described in Supplementary Figures S3 and S4. The differences in baseline characteristics between the training and validation datasets were checked using statistical tests and proved to be insignificant (*p* > 0.05).

### 3.2. Independent Prognostic Variables and Relative Importance

**Figure 2:**
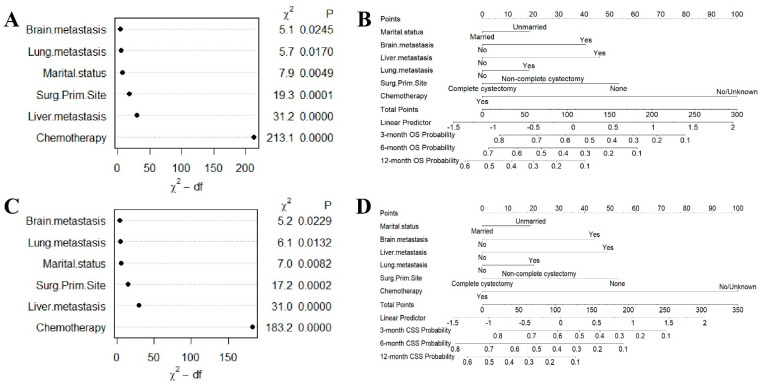
Variable importance and nomograms of (**A**,**B**) overall survival (OS) and (**C**,**D**) cancer-specific survival (CSS).

Supplementary Table S3 showed the details of the univariable Cox regression analysis for OS and CSS. Final variables selected through stepwise multivariable regression analyses are shown in Supplementary Table S4. Results for OS and CSS were consistent. Based on univariable Cox regression, significant factors for OS and CSS were age, marital status, distant metastasis (brain, liver, and lung), primary site surgery, lymph node surgery, and chemotherapy. Multivariable Cox regression analyses (*p* < 0.05) identified marital status, tumor metastasis (brain, liver, and lung), primary site surgery, and chemotherapy as independent factors for OS and CSS, respectively. Regarding the relative importance, chemotherapy ranked highest in contribution measurement, followed by liver metastasis, primary site surgery, marital status, lung metastasis, and brain metastasis (Figure 2A,C).

### 3.3. Construction and Validation of The nomograms

**Figure 3:**
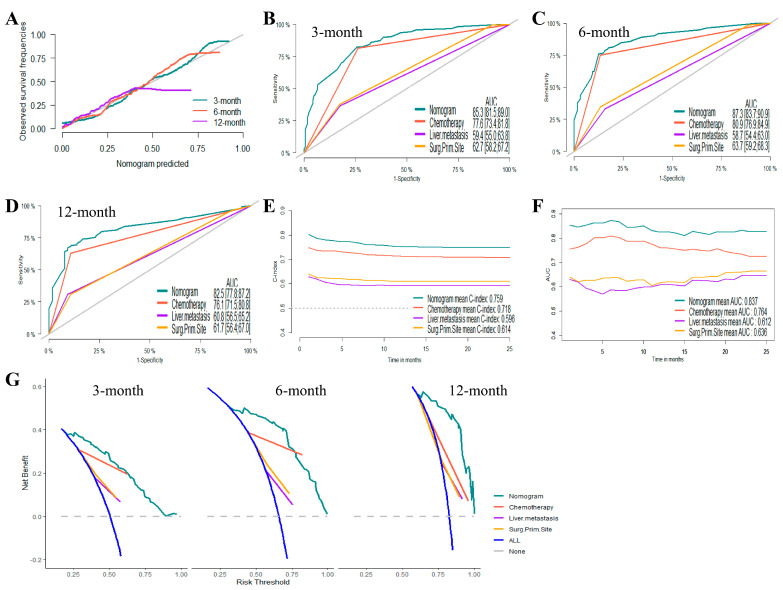
Evaluation of the nomogram on the internal validation dataset for overall survival (OS). (**A**) 3-, 6-, and 12-month calibration plots of nomogram; (**B**) 3-month; (**C**) 6-month; and (**D**) 12-month area under the curve (AUC) for receiver operating characteristic (ROC) curves of nomogram, chemotherapy, liver metastasis, and primary site surgery; (**E**) overall concordance index (c-index) of nomogram, chemotherapy, liver metastasis, and primary site surgery; (**F**) overall AUC of nomogram, chemotherapy, liver metastasis, and primary site surgery; (**G**) 3-, 6-, and 12-month decision curve analysis (DCA) of nomogram, chemotherapy, liver metastasis, and primary site surgery.

**Figure 4:**
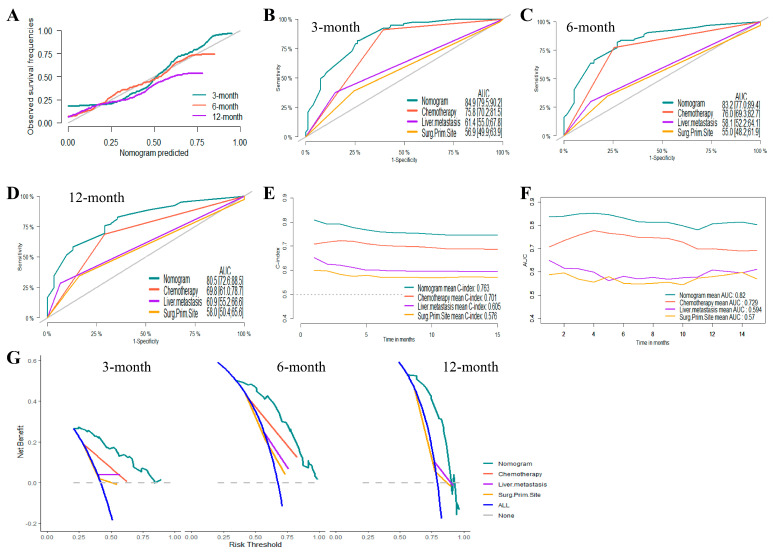
Evaluation of the nomogram on an external testing dataset for overall survival (OS). (**A**) 3-, 6-, and 12-month calibration plots of nomogram; (**B**) 3-month; (**C**) 6-month; and (**D**) 12-month area under the curve (AUC) for receiver operating characteristic (ROC) curves of nomogram, chemotherapy, liver metastasis, and primary site surgery; (**E**) overall concordance index (c-index) of nomogram, chemotherapy, liver metastasis, and primary site surgery; (**F**) Overall AUC of nomogram, chemotherapy, liver metastasis, and primary site surgery; (**G**) 3-, 6-, and 12-month decision curve analysis (DCA) of nomogram, chemotherapy, liver metastasis, and primary site surgery.

**Figure 5:**
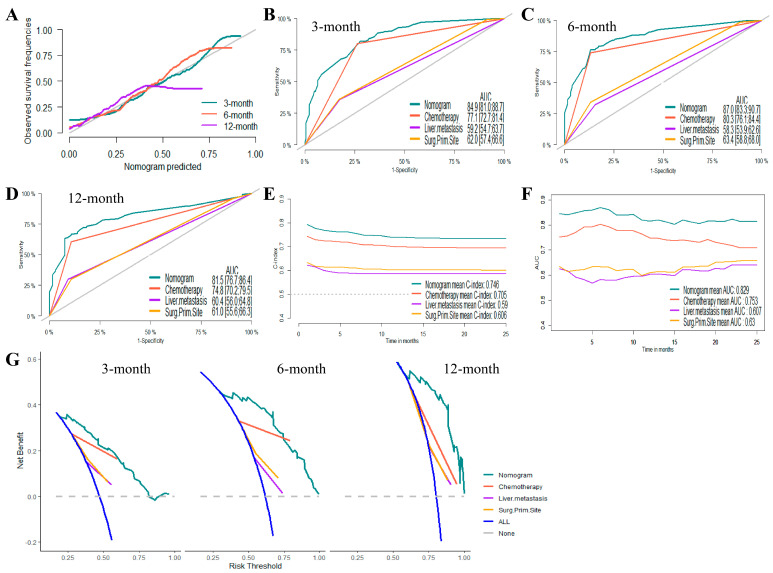
Evaluation of the nomogram on the internal validation dataset for cancer-specific survival (CSS). (**A**) 3-, 6-, and 12-month calibration plots of nomogram. (**B**) 3-month (**C**) 6-month and (**D**) 12-month area under the curve (AUC) for receiver operating characteristic (ROC) curves of nomogram, chemotherapy, liver metastasis, and primary site surgery. (**E**) overall concordance index (c-index) of nomogram, chemotherapy, liver metastasis, and primary site surgery. (**F**) Overall AUC of nomogram, chemotherapy, liver metastasis, and primary site surgery. (**G**) 3-, 6-, and 12-month decision curve analysis (DCA) of nomogram, chemotherapy, liver metastasis, and primary site surgery.

**Figure 6:**
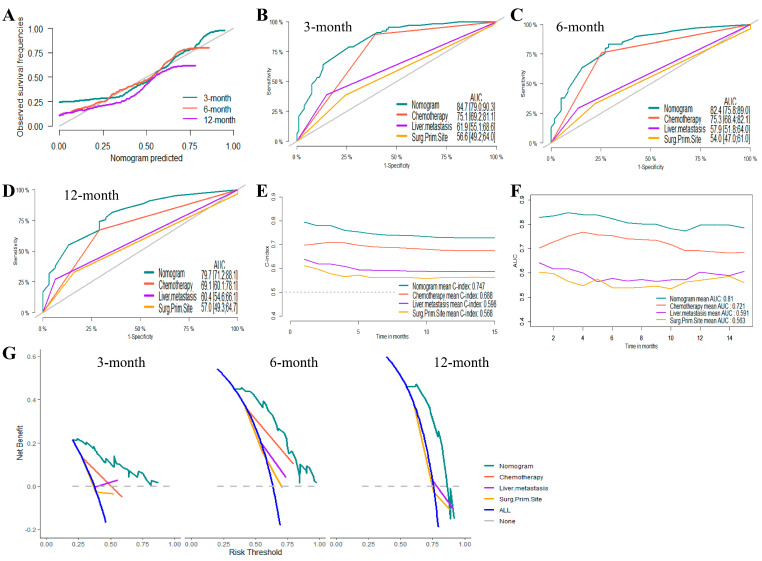
Evaluation of the nomogram on an external testing dataset for cancer-specific survival (CSS). (**A**) 3-, 6-, and 12-month calibration plots of nomogram; (**B**) 3-month; (**C**) 6-month; and (**D**) 12-month area under the curve (AUC) for receiver operating characteristic (ROC) curves of nomogram, Chemotherapy, liver metastasis, and primary site surgery; (**E**) overall concordance index (c-index) of nomogram, chemotherapy, liver metastasis, and primary site surgery; (**F**) Overall AUC of nomogram, chemotherapy, liver metastasis and, primary site surgery; (**G**) 3-, 6-, and 12-month decision curve analysis (DCA) of nomogram, chemotherapy, liver metastasis, and primary site surgery.

Based on the results of the Cox proportional hazards regression model, nomograms were established to predict the 3-month, 6-month, and 12-month OS and CSS, respectively (Figure 2B,D). Supplementary Table S5 showed the scores for each variable in the nomograms. The nomograms for OS and CSS were proved to be accurate, efficient, and clinically beneficial in 3-, 6-, and 12-month survival rate predictions. Calibration plots showed a novel agreement between the observed outcomes and predicted OS (Figure 3A and Figure 4A) or CSS (Figure 5A and Figure 6A) in the internal and external validation. Performances of the top three important single indicators, namely the application of chemotherapy, liver metastasis, and primary site surgery, were measured and compared with nomograms by AUCs and c-indexes (Figure 3, Figure 4, Figure 5 and Figure 6). Nomograms revealed significant advantages over single indicators in OS and CSS prediction, whose AUCs were listed as follows: for OS, 3-month AUCs were 0.853 and 0.849 for validation and testing datasets, respectively (Figure 3B and Figure 4B); 6-month AUCs were 0.873 and 0.832 for validation and testing datasets, respectively (Figure 3C and Figure 4C); 12-month AUCs were 0.825 and 0.805 for validation and testing datasets, respectively (Figure 3D and Figure 4D); for CSS, 3-month AUCs were 0.849 and 0.847 for validation and testing datasets, respectively (Figure 5B and Figure 6B); 6-month AUCs were 0.870 and 0.824 for validation and testing datasets, respectively (Figure 5C and Figure 6C); 12-month AUCs were 0.815 and 0.797 for validation and testing datasets, respectively (Figure 5D and Figure 6D). Table 2 summarizes the sensitivities and specificities of the nomograms in 3-month, 6-month, and 12-month predictions for OS and CSS, which were calculated at the points with the largest Youden indexes. Overall c-indexes distributed by months of follow-up also made it clear that nomograms possessed better discrimination than the three indicators, with mean c-indexes of 0.759 and 0.763 for OS in validation and testing datasets (Figure 3E and Figure 4E), and 0.746 and 0.747 for CSS in validation and testing datasets (Figure 5E and Figure 6E), respectively. The overall AUCs of the nomograms and single indicators are presented in Figure 3, Figure 4, Figure 5 and Figure 6F. To evaluate the clinical applicability of nomograms, DCA curves were conducted and showed that nomograms maximized clinical net benefit under different risk levels and were able to provide positive clinical net benefit for high-risk patients (Figure 3, Figure 4, Figure 5 and Figure 6G). The evaluation of nomograms in the training datasets for OS and CSS are demonstrated in Supplementary Figure S5 and Supplementary Figure S6, respectively. We also evaluated the predictive errors of nomograms with IBS in Supplementary Figure S7. Nomograms showed the lowest predictive errors compared to the three indicators.

### 3.4. Risk Discrimination and Web-Bbased Applications

Patients were divided into two groups (high-risk and low-risk) according to the best cutoff points (142.4 points for OS and 142.5 points for CSS) of the nomograms’ calculated total points determined by X-tile software, as shown in Supplementary Figure S8. KM curves and scatterplots of the risk scores showed that the survival trend prediction of different risk groups was well distinguished in the training (Supplementary Figure S9), internal validation, and testing datasets (Supplementary Figure S10). In addition, we demonstrated subgroup KM survival curves for patients in different risk groups stratified by surgery and chemotherapy status (Supplementary Figures S11 and S12). We found that chemotherapy performed excellent discrimination (log-rank *p* < 0.05) of patients’ survival in different risk groups among the three datasets. Besides, none of the patients in the high-risk group had received chemotherapy. However, the primary site surgery did not show satisfactory stratification in different risk groups. We found that patients with a complete cystectomy had a higher survival probability than those with a non-complete cystectomy or no surgery, in most situations, especially in the high-risk group.

To take advantage of the accuracy, intuitiveness, and clinical benefit of nomograms, we developed online applications for prognostic prediction and clinical decision-making in patients with BCBM (Supplementary Figure S13). The web tools are accessible at https://vincent-y.shinyapps.io/Online_nomogram_for_BCBM_prediction_OS/ (accessed on 1 July 2022.) for OS prediction and https://vincent-y.shinyapps.io/Online_nomogram_for_BCBM_prediction_CSS/ (accessed on 1 July 2022.) for CSS prediction. Examples of hypothetical patients with estimated survival profiles from the online calculators are shown in Supplementary Figures S14 and S15.

## 4. Discussion

To date, few studies have focused on BCBM, and no corresponding predictive model has been developed to evaluate the prognosis of patients with BCBM. In the present study, we used Cox regression analyses to investigate the prognostic factors for OS and CSS, including brain metastasis, lung metastasis, liver metastasis, marital status, chemotherapy, and surgery, and thus developed two nomograms for the prognostic prediction of OS and CSS in BCBM patients. Finally, we constructed two web-based applications, which greatly improved the clinical applicability of the nomograms.

We found that tumor metastatic sites (brain, liver, and lung) were poor prognostic factors for OS and CSS in BCBM patients. This was consistent with previous studies showing multi-site metastases can predict worse OS and CSS [17]. This had also been confirmed in several other metastatic malignancies [18,19]. However, other studies had shown that multi-site metastases was not an independent prognostic factor for OS and CSS [20]. Further studies are needed to elucidate how the number of metastatic sites affects the outcomes of patients with BCBM. Zhang et al. showed that advanced age is a poor prognostic factor in patients with BCBM [21]. However, in our study, age was found to be a prognostic factor in univariate regression for OS and CSS but not in multivariate regression. This may be related to the uneven age distribution of the selected population, as shown in Supplementary Figure S2. We also found that marital status was an independent factor in patients with BCBM, with both OS and CSS being worse in unmarried patients than in married patients. Several mechanisms may explain the association between cancer survival and marital status, which is often considered a marker of social support. Married patients may enjoy more financial resources, have access to social support, have a higher quality of life, and have a healthier lifestyle [22,23]. In addition, married patients could receive better treatment than unmarried patients [24].

In many metastatic urinary or non-urinary cancers, surgical removal of the primary tumor is an integral part of multimodal treatment [25,26]. Our study showed that surgery was an independent factor for OS and CSS in patients with BCBM, which was consistent with previous studies [27,28]. According to previous studies, chemotherapy was considered an important treatment option for metastatic bladder cancer [29]. In our study, chemotherapy was the most important variable in the prognosis prediction of patients with BCBM. Although new advances such as immunotherapy and targeted therapy can improve the survival outcome of patients with metastatic bladder cancer, chemotherapy can still bring survival benefits to patients with BCBM. The subgroup KM survival curves based on chemotherapy also demonstrated that patients who received chemotherapy had a better prognosis than those who did not, as shown in Supplementary Figure S12. The 2018 National Comprehensive Cancer Network (NCCN) guidelines also recommended platinum-based chemotherapy as the standard treatment for patients with metastatic bladder cancer, with an OS of 9 to 15 months [30].

Although studies have reported the impact of these independent prognostic factors, prediction models incorporating these factors into the analysis are lacking. Nomogram has great advantages in integrating different variables that affect the prognosis of patients and has been extensively utilized for the prognostic prediction of patients with malignant tumors [31,32]. The variables used to construct the nomograms in this study included liver metastasis, lung metastasis, brain metastasis, marital status, surgery, and chemotherapy, which can be easily collected in clinical practice. We assessed the performance of nomograms using C-index, calibration curves, and ROC curves and assessed clinical utility using DCA and KM survival curves. The nomograms were proved to be more accurate than the top three indicators in predicting 3-, 6-, and 12-month OS and CSS in the internal and external validation cohorts. In addition, our risk stratification systems divided patients into high- and low-risk groups according to their total nomogram scores, which could help in identifying the high-risk patients, thereby providing accurate therapeutic intervention and monitoring. Furthermore, the web-based nomograms could make it more convenient for the clinical application of our nomograms to assist in treatment decision-making. As demonstrated in Supplementary Figures S14 and S15, patients who received radical cystectomy in combination with chemotherapy had a better 12-month survival prognosis than those who received either radical cystectomy or chemotherapy alone.

However, there are still some limitations to our study. First, some important variables could not be obtained from the SEER database, such as molecular markers, gene expression signatures, information on adjuvant immunotherapy, targeted therapy, systemic therapy, etc., which had been reported in several studies [33,34,35,36,37]. Future studies should focus on integrating multidimensional data to predict the prognosis of BCBM patients, which may improve the practicality and accuracy of the nomograms. Second, multiple imputations were performed for variables with missing values in this study, which might weaken the predictive performance. To minimize this impact, we included only variables with less than 30% missing values, and 100 imputations were executed to improve the robustness. Finally, the datasets we analysed in this study were derived from the single SEER database, although we had performed external validation with the data from 2017. Because the collected population of the SEER dataset is mainly American, validation using data from other countries is required before further application to increase the generalization ability of the models.

## 5. Conclusions

In brief, we comprehensively identified individual prognostic factors for patients with BCBM, including marital status, tumor metastatic sites (brain, liver, and lung), primary site surgery, and chemotherapy. We first established prognostic nomograms for patients with BCBM based on the SEER database. The nomograms were proved to be accurate, efficient, and clinically beneficial. We further developed them as web-based tools to predict OS and CSS for patients with BCBM. These might help in patient management and clinical decision-making for BCBM patients.

## Figures and Tables

**Table 1:** Characteristics of patients in the training dataset, the validation dataset, and the testing dataset.

Characteristics	Level	Whole Dataset (N = 1438)	Internal Validation			External Validation
			Training Dataset (N = 878)	Validation Dataset (N = 376)	*p*-Value	Testing Dataset (N = 184)
Survival, months (mean (SD))		7.89 (10.85)	8.29 (11.11)	7.76 (11.98)	0.45	6.21 (5.92)
OS (%)	Alive	77 (5.4)	34 (3.9)	9 (2.4)	0.25	34 (18.5)
	Dead	1361 (94.6)	844 (96.1)	367 (97.6)		150 (81.5)
CSS (%)	Alive	77 (5.4)	34 (3.9)	9 (2.4)	0.227	34 (18.5)
	Death due to cancer	1274 (88.6)	798 (90.9)	341 (90.7)		135 (73.4)
	Death due to other causes	87 (6.1)	46 (5.2)	26 (6.9)		15 (8.2)
Age (%)	>80	225 (15.6)	142 (16.2)	61 (16.2)	0.572	22 (12.0)
	≤65	597 (41.5)	357 (40.7)	164 (43.6)		76 (41.3)
	65–80	616 (42.8)	379 (43.2)	151 (40.2)		86 (46.7)
Gender (%)	Female	350 (24.3)	224 (25.5)	83 (22.1)	0.22	43 (23.4)
	Male	1088 (75.7)	654 (74.5)	293 (77.9)		141 (76.6)
Race (%)	Black	137 (9.5)	82 (9.3)	44 (11.7)	0.34	11 (6.0)
	Other	81 (5.6)	46 (5.2)	23 (6.1)		12 (6.5)
	White	1220 (84.8)	750 (85.4)	309 (82.2)		161 (87.5)
Marital.status (%)	Married	715 (49.7)	446 (50.8)	175 (46.5)	0.187	94 (51.1)
	Unmarried	723 (50.3)	432 (49.2)	201 (53.5)		90 (48.9)
Histologic.type (%)	Other type	290 (20.2)	179 (20.4)	74 (19.7)	0.842	37 (20.1)
	Papillary transitional cell carcinoma	298 (20.7)	182 (20.7)	74 (19.7)		42 (22.8)
	Transitional cell carcinoma?	850 (59.1)	517 (58.9)	228 (60.6)		105 (57.1)
Grade (%)	Ⅰ	15 (1.0)	8 (0.9)	7 (1.9)	0.507	0 (0.0)
	Ⅱ	74 (5.1)	38 (4.3)	15 (4.0)		21 (11.4)
	Ⅲ	438 (30.5)	283 (32.2)	115 (30.6)		40 (21.7)
	Ⅳ	911 (63.4)	549 (62.5)	239 (63.6)		123 (66.8)
T.stage (%)	T0	18 (1.3)	14 (1.6)	2 (0.5)	0.383	2 (1.1)
	T1	223 (15.5)	135 (15.4)	59 (15.7)		29 (15.8)
	T2	541 (37.6)	341 (38.8)	135 (35.9)		65 (35.3)
	T3	112 (7.8)	69 (7.9)	33 (8.8)		10 (5.4)
	T4	251 (17.5)	156 (17.8)	63 (16.8)		32 (17.4)
	TX	293 (20.4)	163 (18.6)	84 (22.3)		46 (25.0)
N.stage (%)	N0	749 (52.1)	469 (53.4)	190 (50.5)	0.598	90 (48.9)
	N1	136 (9.5)	77 (8.8)	42 (11.2)		17 (9.2)
	N2	243 (16.9)	150 (17.1)	60 (16.0)		33 (17.9)
	N3	95 (6.6)	55 (6.3)	23 (6.1)		17 (9.2)
	NX	215 (15.0)	127 (14.5)	61 (16.2)		27 (14.7)
Brain.metastasis (%)	No	1390 (96.7)	849 (96.7)	360 (95.7)	0.506	181 (98.4)
	Yes	48 (3.3)	29 (3.3)	16 (4.3)		3 (1.6)
Liver.metastasis (%)	No	1090 (75.8)	677 (77.1)	274 (72.9)	0.125	139 (75.5)
	Yes	348 (24.2)	201 (22.9)	102 (27.1)		45 (24.5)
Lung.metastasis (%)	No	1033 (71.8)	633 (72.1)	262 (69.7)	0.425	138 (75.0)
	Yes	405 (28.2)	245 (27.9)	114 (30.3)		46 (25.0)
Surg. Prim. Site (%)	Complete cystectomy	58 (4.0)	37 (4.2)	17 (4.5)	0.955	4 (2.2)
	Non-complete cystectomy	984 (68.4)	604 (68.8)	256 (68.1)		124 (67.4)
	None	396 (27.5)	237 (27.0)	103 (27.4)		56 (30.4)
Surgery.of.lymph.node (%)	No	1361 (94.6)	831 (94.6)	356 (94.7)	1	174 (94.6)
	Yes	77 (5.4)	47 (5.4)	20 (5.3)		10 (5.4)
Radiotherapy (%)	None/Unknown	973 (67.7)	577 (65.7)	271 (72.1)	0.032	125 (67.9)
	Yes	465 (32.3)	301 (34.3)	105 (27.9)		59 (32.1)
Chemotherapy (%)	No/Unknown	756 (52.6)	441 (50.2)	203 (54.0)	0.246	112 (60.9)
	Yes	682 (47.4)	437 (49.8)	173 (46.0)		72 (39.1)

Resection sites for a complete cystectomy are as follows: prostate, seminal vesicles, perivesical tissues, distal ureters, and lymph nodes (male); uterus, ovaries, fallopian tubes, surrounding peritoneum, and lymph nodes (female), which also may include the urethra and vaginal wall.

**Table 2:** Sensitivity and specificity to assess the performance of the nomograms.

Outcome	Dataset	3-Month Prediction		6-Month Prediction		12-Month Prediction	
		Sensitivity	Specificity	Sensitivity	Specificity	Sensitivity	Specificity
Overall survival (OS)	Training dataset	0.827	0.733	0.771	0.729	0.668	0.754
	Validation dataset	0.825	0.743	0.762	0.875	0.691	0.892
	Testing dataset	0.818	0.738	0.824	0.721	0.758	0.701
Cancer-specific survival (CSS)	Training dataset	0.835	0.705	0.746	0.738	0.67	0.726
	Validation dataset	0.837	0.689	0.803	0.838	0.672	0.907
	Testing dataset	0.662	0.847	0.864	0.67	0.774	0.657

## Data Availability

The data analyzed in this study is available on the SEER database (http://seer.cancer.gov/, accessed on 1 July 2022.).

## Supplementary Materials

The following supporting information can be downloaded at: https://www.mdpi.com/article/10.3390/jcm12020726/s1, Supplementary material A: Table S1. Characteristics of patients stratified by survival outcome in whole dataset; Table S2. Characteristics of patients before and after imputation in whole dataset; Table S3. Univariable cox proportion hazards regression analyses for overall survival (OS) and cancer-specific survival (CSS) in training dataset; Table S4. Final selected variables for overall survival (OS) and cancer-specific survival (CSS) in stepwise, backward, multivariable cox proportion hazards regression analyses; Table S5. Score assignment for variables included in the nomograms of overall survival (OS) and cancer-specific survival (CSS); Figure S1. Cumulative incidences curves of cancer and noncancer death in whole dataset; Figure S2. Frequency distribution histogram and density curve of age in whole dataset; Figure S3. Pattern of missing values in whole dataset before imputation; Figure S4. Proportion and combinations of missing values in whole dataset before imputation; Figure S5. Evaluation of the nomogram on training dataset for overall survival (OS). (A) 3-, 6- and 12-month Calibration plots of Nomogram. (B) 3-month (C) 6-month and (D) 12-month Area Under the Curve (AUC) for Receiver Operating Characteristic (ROC) curves of Nomogram, Chemotherapy, Liver metastasis and Primary site surgery. (E) Overall Concordance Index (c-index) of Nomogram, Chemotherapy, Liver metastasis and Primary site surgery. (F) Overall AUC of Nomogram, Chemotherapy, Liver metastasis and Primary site surgery. (G) 3-, 6- and 12-month Decision Curve Analysis (DCA) of Nomogram, Chemotherapy, Liver metastasis and Primary site surgery; Figure S6. Evaluation of the nomogram on training dataset for cancer-specific survival (CSS). (A) 3-, 6- and 12-month Calibration plots of Nomogram. (B) 3-month (C) 6-month and (D) 12-month Area Under the Curve (AUC) for Receiver Operating Characteristic (ROC) curves of Nomogram, Chemotherapy, Liver metastasis and Primary site surgery. (E) Overall Concordance Index (c-index) of Nomogram, Chemotherapy, Liver metastasis and Primary site surgery. (F) Overall AUC of Nomogram, Chemotherapy, Liver metastasis and Primary site surgery. (G) 3-, 6- and 12-month Decision Curve Analysis (DCA) of Nomogram, Chemotherapy, Liver metastasis and Primary site surgery; Figure S7. The prediction error curves for different models based on the integrated Brier score (IBS). (A) Training dataset of overall survival (OS). (B) Internal validation dataset of overall survival (OS). (C) External testing dataset of overall survival (OS). (D) Training dataset of cancer-specific survival (CSS). (E) Internal validation dataset of cancer-specific survival (CSS). (F) External testing dataset of cancer-specific survival (CSS); Figure S8. Cut-off values of nomogram total points calculated by X-tile. (A) Training dataset of Overall survival (OS). (B) Training dataset of cancer-specific survival (CSS); Figure S9. Kaplan-Meier survival curves of patients stratified by risk score and scatterplot of the risk score for (A, C) Training dataset of overall survival (OS). (B, D) Training dataset of cancer-specific survival (CSS); Figure S10. Kaplan-Meier survival curves of patients stratified by risk score and scatterplot of the risk score for (A, E) Internal validation of overall survival (OS). (B, F) External testing dataset of overall survival (OS). (C, G) Internal validation dataset of cancer-specific survival (CSS). (D, H) External testing dataset of cancer-specific survival (CSS); Figure S11. Kaplan–Meier curves of patients with different surgery in low- (A-C) and high-risk (D-F) group for Overall survival (OS) and low- (G-I) and high-risk (J-L) group for Cancer-specific survival (CSS); Figure S12. Kaplan–Meier curves of patients with different chemotherapy in low- (A-C) and high-risk (D-F) group for Overall survival (OS) and low- (G-I) and high-risk (J-L) group for Cancer-specific survival (CSS); Figure S13. The web survival rate calculator for (A) Overall survival (OS). (B) Cancer-specific survival (CSS); Figure S14. The web survival rate calculator estimated overall survival (OS) of a hypothetical patient (Married; No brain metastasis, liver, or lung metastasis) based on different treatment strategy. (A) Survival curves of different treatments. (B) 12-month survival probability with 95%CI of different treatments. Abbreviations: CC, complete cystectomy; CT, chemotherapy; Figure S15. The web survival rate calculator estimated cancer-specific survival (CSS) of a hypothetical patient (Married; No brain metastasis, liver, or lung metastasis) based on different treatment strategy. (A) Survival curves of different treatments. (B) 12-month survival probability with 95%CI of different treatments. Abbreviations: CC, complete cystectomy; CT, chemotherapy; Supplementary material B: STROBE Statement for Checklist of items that should be included in reports of cohort studies; Supplementary material C: TRIPOD Checklist for Prediction Model Development and Validation.

## Abbreviations

BCa: bladder cancer; mBC, metastatic bladder cancer; SREs, skeletal related events; BCBM, bladder cancer with bone metastases; SEER, surveillance, epidemiology, and end results; OS, overall survival; CSS, cancer-specific survival; ICD-O-3, Third Edition of International Classification of Diseases for Oncology; HR, hazard ratio; CI, confidence interval; KM, Kaplan–Meier; AUC; area under the curve; ROC, receiver operating characteristic; C-index, concordance Index; DCA, decision curve analysis; IBS, integrated Brier score; NCCN, National Comprehensive Cancer Network.
