# Supplementary material for: Web-Based Nomograms for Overall Survival and Cancer-Specific Survival of Bladder Cancer Patients with Bone Metastasis: A Retrospective Cohort Study from SEER Database
Source: J Clin Med. 2023 Jan 16;12(2):726. doi: 10.3390/jcm12020726 (PMC9865586; doi:10.3390/jcm12020726)
Supplement: Supplementary file 1 [file jcm-12-00726-s001.zip › jcm-2151781-supplementary.pdf]

Supplementary contents

Supplementary material A

Supplementary Table S1. Characteristics of patients stratified by survival outcome in whole dataset.

Supplementary Table S2. Characteristics of patients before and after imputation in whole dataset.

Supplementary Table S3. Univariable cox proportion hazards regression analyses for overall survival (OS) and cancer-specific survival (CSS) in training dataset.

Supplementary Table S4. Final selected variables for overall survival (OS) and cancer-specific survival (CSS) in stepwise, backward, multivariable cox proportion hazards regression analyses.

Supplementary Table S5. Score assignment for variables included in the nomograms of overall survival (OS) and cancer-specific survival (CSS).

Supplementary Figure S1. Cumulative incidences curves of cancer and noncancer death in whole dataset.

Supplementary Figure S2. Frequency distribution histogram and density curve of age in whole dataset.

Supplementary Figure S3. Pattern of missing values in whole dataset before imputation.

Supplementary Figure S4. Proportion and combinations of missing values in whole dataset before imputation.

Supplementary Figure S5. Evaluation of the nomogram on training dataset for overall survival (OS). (A) 3-, 6- and 12-month Calibration plots of Nomogram. (B) 3-month (C) 6-month and (D) 12-month Area Under the Curve (AUC) for Receiver Operating Characteristic (ROC) curves of Nomogram, Chemotherapy, Liver metastasis and Primary site surgery. (E) Overall Concordance Index (c-index) of Nomogram, Chemotherapy, Liver metastasis and Primary site surgery. (F) Overall AUC of Nomogram, Chemotherapy, Liver metastasis and Primary site surgery. (G) 3-, 6- and 12-month Decision Curve Analysis (DCA) of Nomogram, Chemotherapy, Liver metastasis and Primary site surgery.

Supplementary Figure S6. Evaluation of the nomogram on training dataset for cancer-specific survival (CSS). (A) 3-, 6- and 12-month Calibration plots of Nomogram. (B) 3-month (C) 6-month and (D) 12-month Area Under the Curve (AUC) for Receiver Operating Characteristic (ROC) curves of Nomogram, Chemotherapy, Liver metastasis and Primary site surgery. (E) Overall Concordance Index (c-index) of Nomogram, Chemotherapy, Liver metastasis and Primary site surgery. (F) Overall AUC of Nomogram, Chemotherapy, Liver metastasis and Primary site surgery. (G) 3-, 6- and 12-month Decision Curve Analysis (DCA) of Nomogram, Chemotherapy, Liver metastasis and Primary site surgery.

Supplementary Figure S7. The prediction error curves for different models based on the integrated Brier score (IBS). (A) Training dataset of overall survival (OS). (B) Internal validation dataset of overall survival (OS). (C) External testing dataset of overall survival (OS). (D) Training dataset of cancer-specific survival (CSS). (E) Internal validation dataset of cancer-specific survival (CSS). (F) External testing dataset of cancer-specific survival (CSS).

Supplementary Figure S8. Cut-off values of nomogram total points calculated by X-tile. (A) Training dataset of Overall survival (OS). (B) Training dataset of cancer-specific survival (CSS).

Supplementary Figure S9. Kaplan-Meier survival curves of patients stratified by risk score and scatterplot of the risk score for (A, C) Training dataset of overall survival (OS). (B, D) Training dataset of cancer-specific survival (CSS).

Supplementary Figure S10. Kaplan-Meier survival curves of patients stratified by risk score and scatterplot of the risk score for (A, E) Internal validation of overall survival (OS). (B, F) External testing dataset of overall survival (OS). (C, G) Internal validation dataset of cancer-specific survival (CSS). (D, H) External testing dataset of cancer-specific survival (CSS).

Supplementary Figure S11. Kaplan–Meier curves of patients with different surgery in low- (A-C) and high-risk (D-F) group for Overall survival (OS) and low- (G-I) and high-risk (J-L) group for Cancer-specific survival (CSS).

Supplementary Figure S12. Kaplan–Meier curves of patients with different chemotherapy in low- (A-C) and high-risk (D-F) group for Overall survival (OS) and low- (G-I) and high-risk (J-L) group for Cancer-specific survival (CSS).

Supplementary Figure S13. The web survival rate calculator for (A) Overall survival (OS). (B) Cancer-specific survival (CSS).

Supplementary Figure S14. The web survival rate calculator estimated overall survival (OS) of a hypothetical patient (Married; No brain metastasis, liver or lung metastasis) based on different treatment strategy. (A) Survival curves of different treatments. (B) 12-month survival probability with 95%CI of different treatments. Abbreviations: CC, complete cystectomy; CT, chemotherapy.

Supplementary Figure S15. The web survival rate calculator estimated cancer-specific survival (CSS) of a hypothetical patient (Married; No brain metastasis, liver or lung metastasis) based on different treatment strategy. (A) Survival curves of different treatments. (B) 12-month survival probability with 95%CI of different treatments. Abbreviations: CC, complete cystectomy; CT, chemotherapy.

Supplementary material B
STROBE Statement for Checklist of items that should be included in reports of cohort studies

Supplementary material C
TRIPOD Checklist for Prediction Model Development and Validation

Supplementary material A

Supplementary Table S1. Characteristics of patients stratified by survival outcome in whole dataset.

Characteristics	Level	Overall (N = 1438)	Alive (N = 77)	Dead of cancer (N = 1274)	Dead of other causes (N = 87)	*p* value
Survival.months (mean (SD))		7.89 (10.85)	28.68 (21.31)	6.62 (8.03)	8.10 (14.19)	<0.001
OS (%)	Alive	77 (5.4)	77 (100.0)	0 (0.0)	0 (0.0)	<0.001
	Dead	1361 (94.6)	0 (0.0)	1274 (100.0)	87 (100.0)	
Year.of.diagnosis (%)	2010	159 (11.1)	1 (1.3)	148 (11.6)	10 (11.5)	<0.001
	2011	149 (10.4)	1 (1.3)	141 (11.1)	7 (8.0)	
	2012	192 (13.4)	3 (3.9)	174 (13.7)	15 (17.2)	
	2013	162 (11.3)	1 (1.3)	151 (11.9)	10 (11.5)	
	2014	174 (12.1)	10 (13.0)	158 (12.4)	6 (6.9)	
	2015	198 (13.8)	10 (13.0)	175 (13.7)	13 (14.9)	
	2016	220 (15.3)	17 (22.1)	192 (15.1)	11 (12.6)	
	2017	184 (12.8)	34 (44.2)	135 (10.6)	15 (17.2)	
Age (%)	＞80	225 (15.6)	5 (6.5)	201 (15.8)	19 (21.8)	0.113
	≤65	597 (41.5)	36 (46.8)	528 (41.4)	33 (37.9)	
	65-80	616 (42.8)	36 (46.8)	545 (42.8)	35 (40.2)	
Gender (%)	Female	350 (24.3)	18 (23.4)	309 (24.3)	23 (26.4)	0.882
	Male	1088 (75.7)	59 (76.6)	965 (75.7)	64 (73.6)	
Race (%)	Black	137 (9.5)	10 (13.0)	109 (8.6)	18 (20.7)	0.002
	Other	81 (5.6)	7 (9.1)	69 (5.4)	5 (5.7)	
	White	1220 (84.8)	60 (77.9)	1096 (86.0)	64 (73.6)	
Marital.status (%)	Married	715 (49.7)	52 (67.5)	629 (49.4)	34 (39.1)	0.001
	Unmarried	723 (50.3)	25 (32.5)	645 (50.6)	53 (60.9)	
Histologic.type (%)	Other type	290 (20.2)	13 (16.9)	250 (19.6)	27 (31.0)	0.001
	Papillary transitional cell carcinoma	298 (20.7)	28 (36.4)	257 (20.2)	13 (14.9)	
	Transitional cell carcinoma?	850 (59.1)	36 (46.8)	767 (60.2)	47 (54.0)	
Grade (%)	Ⅰ	15 (1.0)	0 (0.0)	12 (0.9)	3 (3.4)	0.007
	Ⅱ	74 (5.1)	8 (10.4)	61 (4.8)	5 (5.7)	
	Ⅲ	438 (30.5)	12 (15.6)	398 (31.2)	28 (32.2)	
	Ⅳ	911 (63.4)	57 (74.0)	803 (63.0)	51 (58.6)	
T.stage (%)	T0	18 (1.3)	1 (1.3)	15 (1.2)	2 (2.3)	0.024
	T1	223 (15.5)	18 (23.4)	192 (15.1)	13 (14.9)	
	T2	541 (37.6)	37 (48.1)	475 (37.3)	29 (33.3)	
	T3	112 (7.8)	8 (10.4)	99 (7.8)	5 (5.7)	
	T4	251 (17.5)	9 (11.7)	228 (17.9)	14 (16.1)	
	TX	293 (20.4)	4 (5.2)	265 (20.8)	24 (27.6)	
N.stage (%)	N0	749 (52.1)	43 (55.8)	661 (51.9)	45 (51.7)	0.215
	N1	136 (9.5)	10 (13.0)	117 (9.2)	9 (10.3)	
	N2	243 (16.9)	9 (11.7)	225 (17.7)	9 (10.3)	

	N3	95 (6.6)	8 (10.4)	81 (6.4)	6 (6.9)	
	NX	215 (15.0)	7 (9.1)	190 (14.9)	18 (20.7)	
Brain.metastasis (%)	No	1390 (96.7)	77 (100.0)	1230 (96.5)	83 (95.4)	0.208
	Yes	48 (3.3)	0 (0.0)	44 (3.5)	4 (4.6)	
Liver.metastasis (%)	No	1090 (75.8)	70 (90.9)	955 (75.0)	65 (74.7)	0.006
	Yes	348 (24.2)	7 (9.1)	319 (25.0)	22 (25.3)	
Lung.metastasis (%)	No	1033 (71.8)	66 (85.7)	903 (70.9)	64 (73.6)	0.018
	Yes	405 (28.2)	11 (14.3)	371 (29.1)	23 (26.4)	
Surg.Prim.Site (%)	Complete cystectomy	58 (4.0)	6 (7.8)	51 (4.0)	1 (1.1)	0.003
	Non-complete cystectomy	984 (68.4)	63 (81.8)	864 (67.8)	57 (65.5)	
	None	396 (27.5)	8 (10.4)	359 (28.2)	29 (33.3)	
Surgery.of.lymph.node (%)	No	1361 (94.6)	65 (84.4)	1210 (95.0)	86 (98.9)	<0.001
	Yes	77 (5.4)	12 (15.6)	64 (5.0)	1 (1.1)	
Radiotherapy (%)	None/Unknown	973 (67.7)	54 (70.1)	855 (67.1)	64 (73.6)	0.412
	Yes	465 (32.3)	23 (29.9)	419 (32.9)	23 (26.4)	
Chemotherapy (%)	No/Unknown	756 (52.6)	21 (27.3)	673 (52.8)	62 (71.3)	<0.001
	Yes	682 (47.4)	56 (72.7)	601 (47.2)	25 (28.7)	

Supplementary Table S2. Characteristics of patients before and after imputation in whole dataset.

Characteristics	Level	Before imputation (N = 1438)	After imputation (N = 1438)	*p* value
Survival.months (mean (SD))		7.89 (10.85)	7.89 (10.85)	1
OS (%)	Alive	77 (5.4)	77 (5.4)	1
	Dead	1361 (94.6)	1361 (94.6)	
CSS (%)	Alive	77 (5.4)	77 (5.4)	1
	Dead of cancer	1274 (88.6)	1274 (88.6)	
	Dead of other causes	87 (6.1)	87 (6.1)	
Year.of.diagnosis (%)	2010	159 (11.1)	159 (11.1)	1
	2011	149 (10.4)	149 (10.4)	
	2012	192 (13.4)	192 (13.4)	
	2013	162 (11.3)	162 (11.3)	
	2014	174 (12.1)	174 (12.1)	
	2015	198 (13.8)	198 (13.8)	
	2016	220 (15.3)	220 (15.3)	
	2017	184 (12.8)	184 (12.8)	
Age (%)	＞80	225 (15.6)	225 (15.6)	1
	≤65	597 (41.5)	597 (41.5)	
	65-80	616 (42.8)	616 (42.8)	
Gender (%)	Female	350 (24.3)	350 (24.3)	1
	Male	1088 (75.7)	1088 (75.7)	
Race (%)	Black	137 (9.5)	137 (9.5)	1
	Other	81 (5.6)	81 (5.6)	
	White	1219 (84.8)	1220 (84.8)	
Marital.status (%)	Married	681 (49.8)	715 (49.7)	1
	Unmarried	687 (50.2)	723 (50.3)	
Histologic.type (%)	Other type	290 (20.2)	290 (20.2)	1
	Papillary transitional cell carcinoma	298 (20.7)	298 (20.7)	
	Transitional cell carcinoma?	850 (59.1)	850 (59.1)	
Grade (%)	Ⅰ	9 (0.8)	15 (1.0)	0.028
	Ⅱ	48 (4.5)	74 (5.1)	
	Ⅲ	271 (25.5)	438 (30.5)	
	Ⅳ	734 (69.1)	911 (63.4)	
T.stage (%)	T0	18 (1.3)	18 (1.3)	1
	T1	221 (15.5)	223 (15.5)	
	T2	538 (37.6)	541 (37.6)	
	T3	112 (7.8)	112 (7.8)	
	T4	250 (17.5)	251 (17.5)	
	TX	290 (20.3)	293 (20.4)	
N.stage (%)	N0	747 (52.3)	749 (52.1)	1
	N1	134 (9.4)	136 (9.5)	
	N2	241 (16.9)	243 (16.9)	

	N3	95 (6.6)	95 (6.6)	
	NX	212 (14.8)	215 (15.0)	
Brain.metastasis (%)	No	1351 (96.6)	1390 (96.7)	1
	Yes	47 (3.4)	48 (3.3)	
Liver.metastasis (%)	No	1073 (76.0)	1090 (75.8)	0.94
	Yes	339 (24.0)	348 (24.2)	
Lung.metastasis (%)	No	1003 (71.8)	1033 (71.8)	1
	Yes	394 (28.2)	405 (28.2)	
Surg.Prim.Site (%)	Complete cystectomy	58 (4.0)	58 (4.0)	1
	Non-complete cystectomy	983 (68.5)	984 (68.4)	
	None	395 (27.5)	396 (27.5)	
Surgery.of.lymph.node (%)	No	1356 (94.6)	1361 (94.6)	1
	Yes	77 (5.4)	77 (5.4)	
Radiotherapy (%)	None/Unknown	973 (67.7)	973 (67.7)	1
	Yes	465 (32.3)	465 (32.3)	
Chemotherapy (%)	No/Unknown	756 (52.6)	756 (52.6)	1
	Yes	682 (47.4)	682 (47.4)	

Supplementary Table S3. Univariable cox proportion hazards regression analyses for overall survival (OS) and cancer-specific survival (CSS) in training dataset.

Variables	Overall survival (OS)		Cancer-specific survival (CSS)	
	HR (95%CI)	*p* value	HR (95%CI)	*p* value
Age ≤ 65	0.68 (0.56-0.83)	<0.001	0.72 (0.58-0.88)	0.002
Age65-80	0.77 (0.63-0.93)	0.008	0.8 (0.66-0.99)	0.037
GenderMale	0.87 (0.75-1.02)	0.087	0.86 (0.73-1.01)	0.065
RaceBlack	1.11 (0.76-1.61)	0.601	0.94 (0.64-1.39)	0.77
RaceWhite	1.12 (0.82-1.53)	0.466	1.09 (0.8-1.5)	0.58
Marital.statusUnmarried	1.38 (1.2-1.58)	<0.001	1.35 (1.18-1.56)	<0.001
Histologic.typePapillary transitional cell carcinoma	0.9 (0.73-1.11)	0.338	0.92 (0.74-1.14)	0.45
Histologic.typeTransitional cell carcinoma?	1.05 (0.88-1.24)	0.612	1.07 (0.9-1.28)	0.448
Grade Ⅱ	0.89 (0.41-1.93)	0.763	0.98 (0.43-2.23)	0.965
GradeⅢ	1.13 (0.55-2.3)	0.737	1.16 (0.54-2.47)	0.709
GradeⅣ	0.9 (0.45-1.83)	0.781	0.95 (0.45-2.02)	0.891
T.stageT1	0.84 (0.48-1.46)	0.53	0.93 (0.51-1.69)	0.82
T.stageT2	0.78 (0.46-1.34)	0.368	0.86 (0.48-1.53)	0.6
T.stageT3	0.7 (0.39-1.24)	0.222	0.78 (0.42-1.45)	0.441
T.stageT4	0.92 (0.53-1.6)	0.772	1.02 (0.57-1.85)	0.942
T.stageTX	1.18 (0.68-2.05)	0.545	1.28 (0.71-2.3)	0.42
N.stageN1	0.92 (0.71-1.17)	0.487	0.91 (0.7-1.17)	0.459
N.stageN2	1.05 (0.87-1.27)	0.589	1.09 (0.9-1.32)	0.364
N.stageN3	1 (0.75-1.33)	0.975	1.04 (0.78-1.39)	0.791
N.stageNX	1.06 (0.87-1.29)	0.565	1.01 (0.82-1.24)	0.95
Brain.metastasisYes	1.84 (1.27-2.66)	0.001	1.86 (1.28-2.72)	0.001
Liver.metastasisYes	1.7 (1.44-1.99)	<0.001	1.73 (1.47-2.04)	<0.001
Lung.metastasisYes	1.37 (1.18-1.59)	<0.001	1.4 (1.2-1.63)	<0.001
Surg.Prim.SiteComplete cystectomy	0.41 (0.28-0.59)	<0.001	0.42 (0.29-0.62)	<0.001
Surg.Prim.SiteNon-complete cystectomy	0.65 (0.56-0.76)	<0.001	0.65 (0.56-0.76)	<0.001
Surgery.of.lymph.nodeYes	0.54 (0.4-0.74)	<0.001	0.57 (0.41-0.78)	0.001
RadiotherapyYes	0.99 (0.86-1.14)	0.92	1.01 (0.87-1.17)	0.903
ChemotherapyYes	0.34 (0.29-0.39)	<0.001	0.36 (0.31-0.41)	<0.001

Supplementary Table S4. Final selected variables for overall survival (OS) and cancer-specific survival (CSS) in stepwise, backward, multivariable cox proportion hazards regression analyses.

Variables	Overall survival (OS)			Cancer-specific survival (CSS)		
	**β- coefficient**	**HR (95%CI)**	***p* value**	**β- coefficient**	**HR (95%CI)**	***p* value**
Marital.status						
Married	Reference			Reference		
Unmarried	0.199	1.22 (1.06-1.4)	0.0049	0.192	1.21 (1.05-1.4)	0.0082
Brain.metastasis						
No	Reference			Reference		
Yes	0.431	1.54 (1.06-2.24)	0.0249	0.444	1.56 (1.06-2.29)	0.0233
Liver.metastasis						
No	Reference			Reference		
Yes	0.489	1.63 (1.37-1.94)	<0.001	0.499	1.65 (1.38-1.96)	<0.001
Lung.metastasis						
No	Reference			Reference		
Yes	0.194	1.21 (1.04-1.42)	0.017	0.206	1.23 (1.04-1.45)	0.0132
Surg.Prim.Site						
None	Reference			Reference		
Complete cystectomy	-0.57	0.57 (0.39-0.82)	0.0025	-0.537	0.58 (0.4-0.85)	0.0051
Non-complete cystectomy	-0.318	0.73 (0.62-0.85)	<0.001	-0.311	0.73 (0.62-0.86)	<0.001
Chemotherapy						
No/Unknown	Reference			Reference		
Yes	-1.064	0.35 (0.3-0.4)	<0.001	-1.014	0.36 (0.31-0.42)	<0.001

Supplementary Table S5. Score assignment for variables included in the nomograms of overall survival (OS) and cancer-specific survival (CSS).

Overall survival (OS)			Cancer-specific survival (CSS)		
**Variables**	**Category**	**Score**	**Variables**	**Category**	**Score**
**Marital status**	Married	0	**Marital status**	Married	0
	Unmarried	19		Unmarried	19
**Brain metastasis**	No	0	**Brain metastasis**	No	0
	Yes	41		Yes	44
**Liver metastasis**	No	0	**Liver metastasis**	No	0
	Yes	46		Yes	49
**Lung metastasis**	No	0	**Lung metastasis**	No	0
	Yes	18		Yes	20
**Surg Prim Site**	Complete cystectomy	0	**Surg Prim Site**	Complete cystectomy	0
	Non-complete cystectomy	24		Non-complete cystectomy	22
	None	54		None	53
**Chemotherapy**	No/Unknown	100	**Chemotherapy**	No/Unknown	100
	Yes	0		Yes	0
**3-month Survival**			**3-month Survival**		
**Total scores**	**3-month Survival Probability**		**Total scores**	**3-month Survival Probability**	
239	0.1		252	0.1	
206	0.2		216	0.2	
178	0.3		188	0.3	
153	0.4		161	0.4	
127	0.5		133	0.5	
98	0.6		103	0.6	
64	0.7		68	0.7	
20	0.8		22	0.8	
**6-month Survival**			**6-month Survival**		
**Total scores**	**6-month Survival Probability**		**Total scores**	**6-month Survival Probability**	
182	0.1		193	0.1	
149	0.2		158	0.2	
121	0.3		129	0.3	
96	0.4		102	0.4	
70	0.5		75	0.5	
41	0.6		45	0.6	
7	0.7		9	0.7	
			-37	0.8	
**12-month Survival**			**12-month Survival**		

Total scores	12-month Survival Probability	Total scores	12-month Survival Probability
121	0.1	128	0.1
87	0.2	93	0.2
60	0.3	64	0.3
35	0.4	37	0.4
8	0.5	10	0.5
-20	0.6	-20	0.6

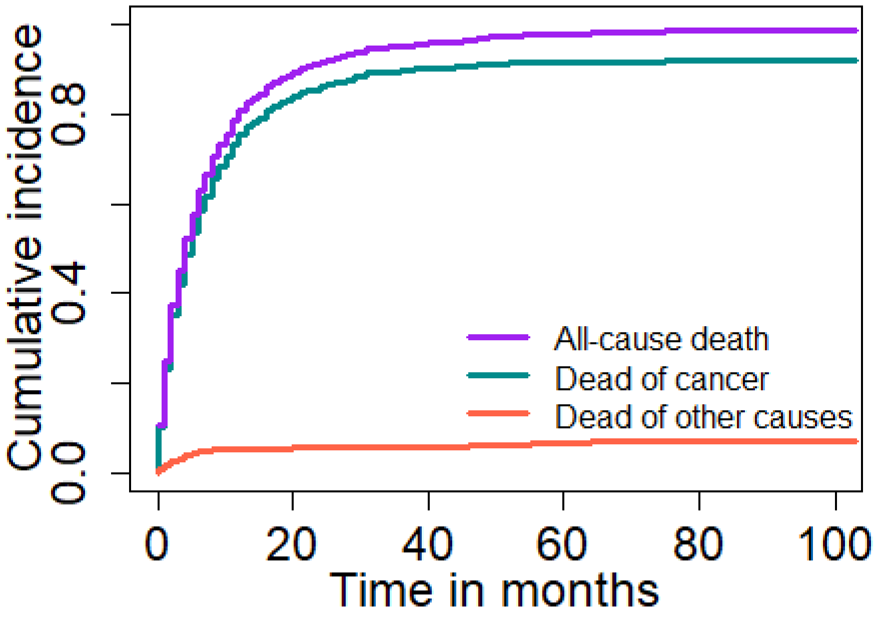

Supplementary Figure S1. Cumulative incidences curves of cancer and noncancer death in whole dataset.

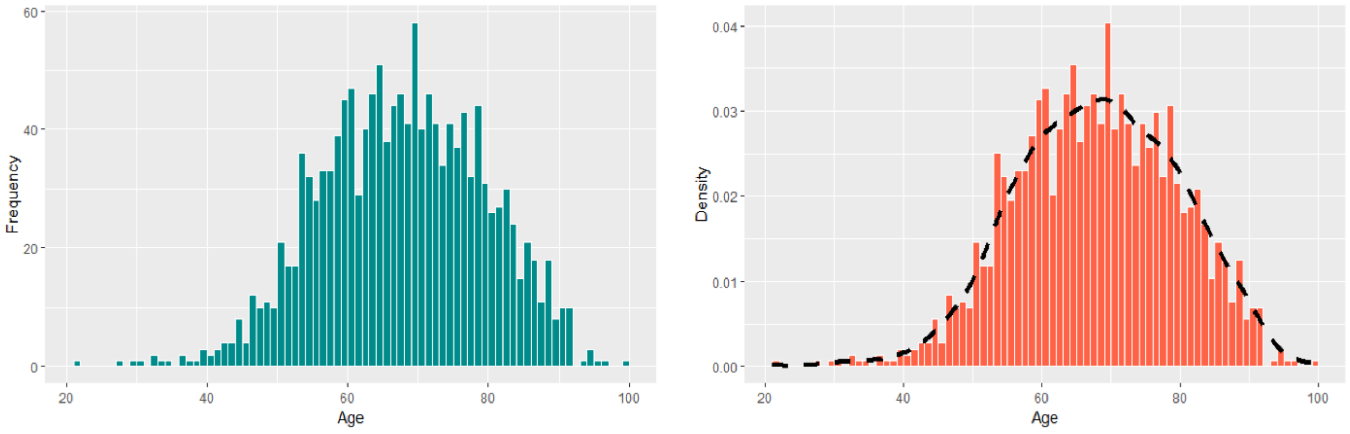

Supplementary Figure S2. Frequency distribution histogram and density curve of age in whole dataset.

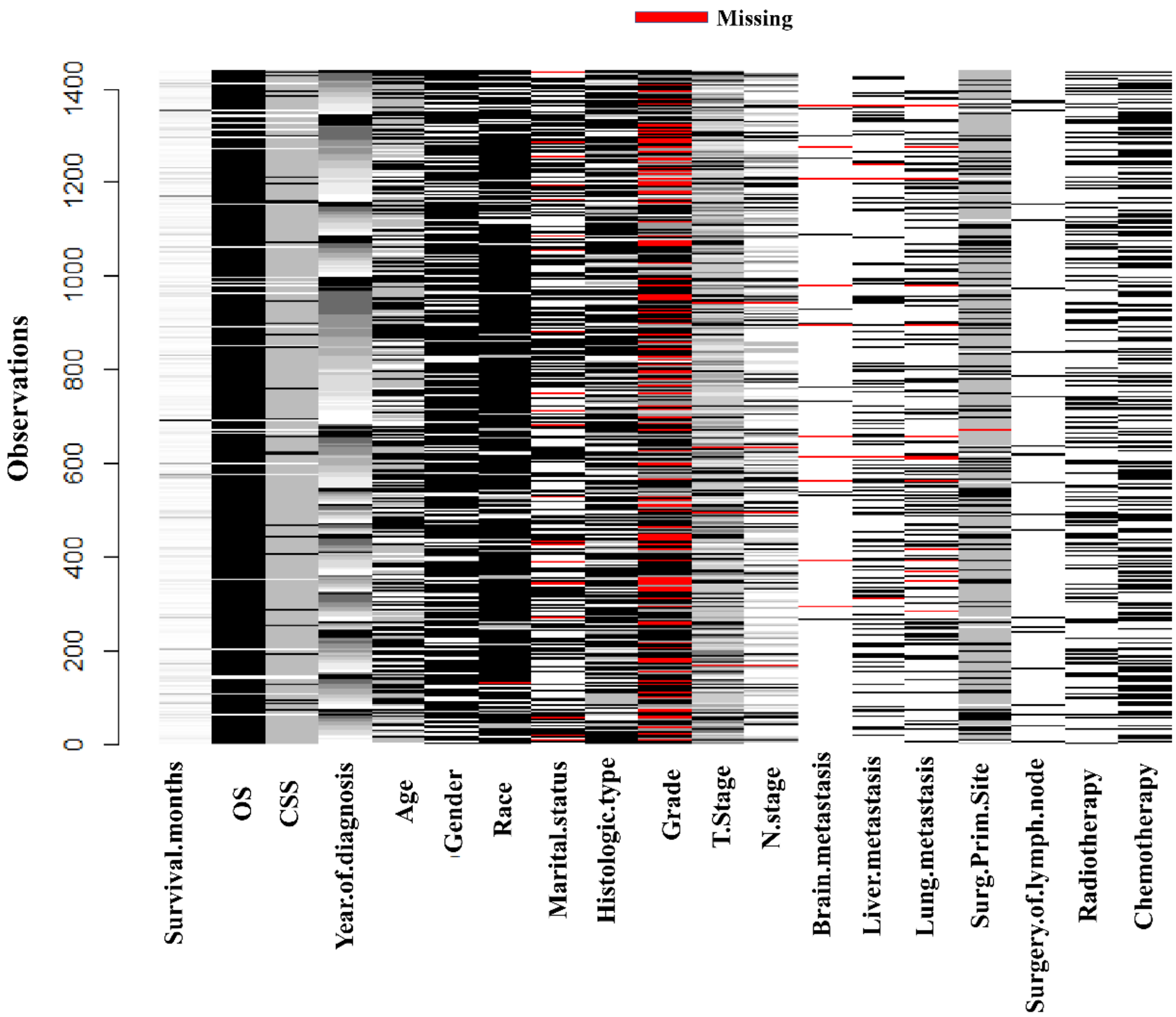

Supplementary Figure S3. Pattern of missing values in whole dataset before imputation.

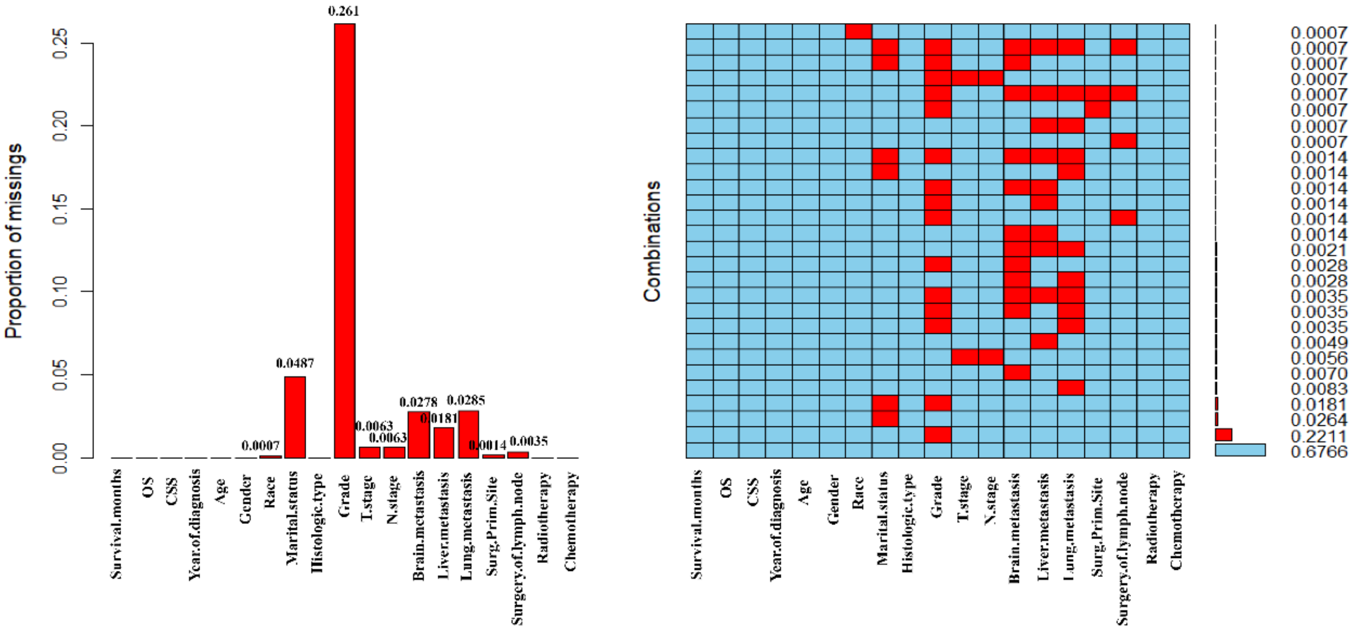

Supplementary Figure S4. Proportion and combinations of missing values in whole dataset before imputation.

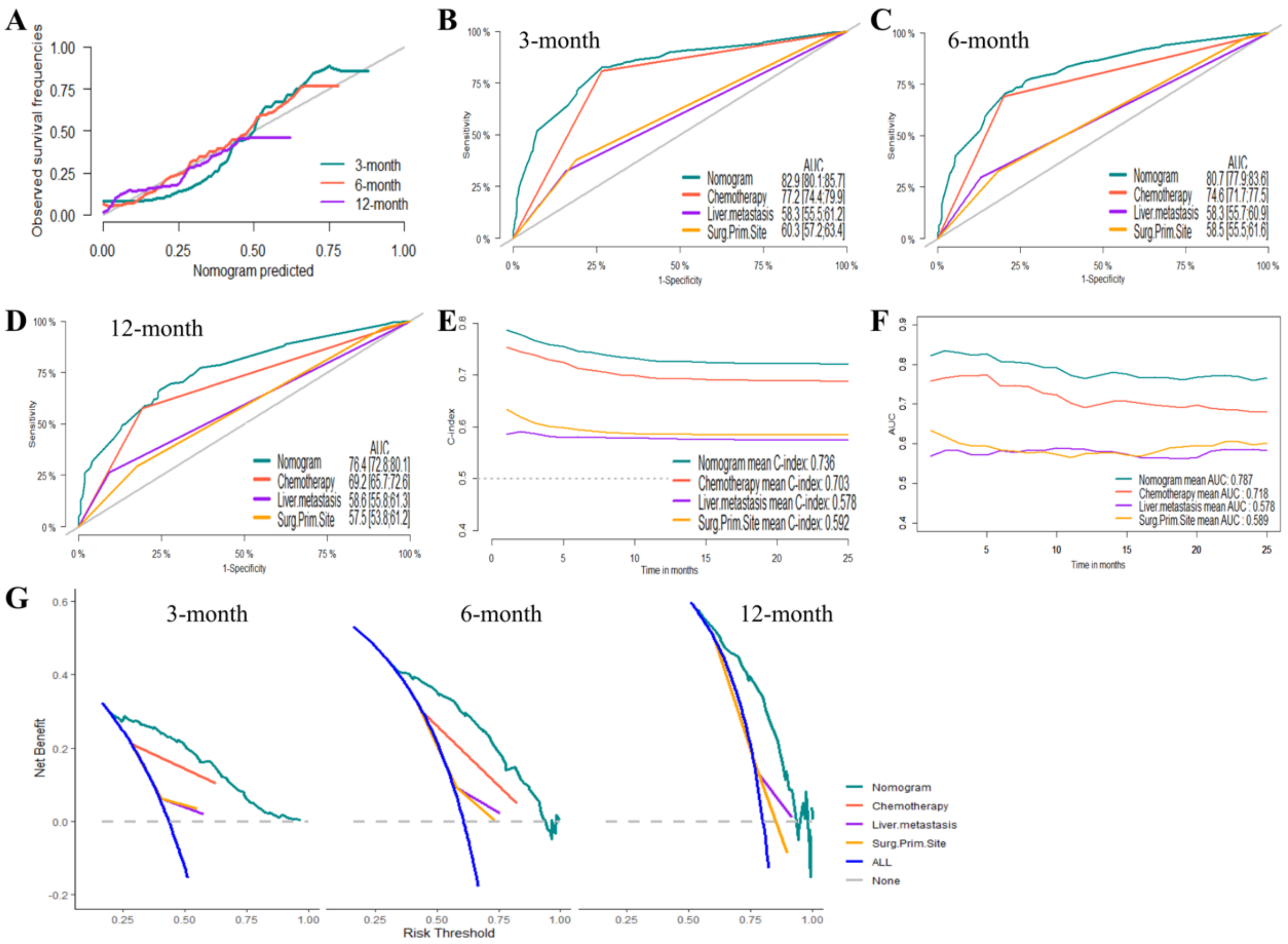

Supplementary Figure S5. Evaluation of the nomogram on training dataset for overall survival (OS). (A) 3-, 6- and 12-month Calibration plots of Nomogram. (B) 3-month (C) 6-month and (D) 12-month Area Under the Curve (AUC) for Receiver Operating Characteristic (ROC) curves of Nomogram, Chemotherapy, Liver metastasis and Primary site surgery. (E) Overall Concordance Index (c-index) of Nomogram, Chemotherapy, Liver metastasis and Primary site surgery. (F) Overall AUC of Nomogram, Chemotherapy, Liver metastasis and Primary site surgery. (G) 3-, 6- and 12-month Decision Curve Analysis (DCA) of Nomogram, Chemotherapy, Liver metastasis and Primary site surgery.

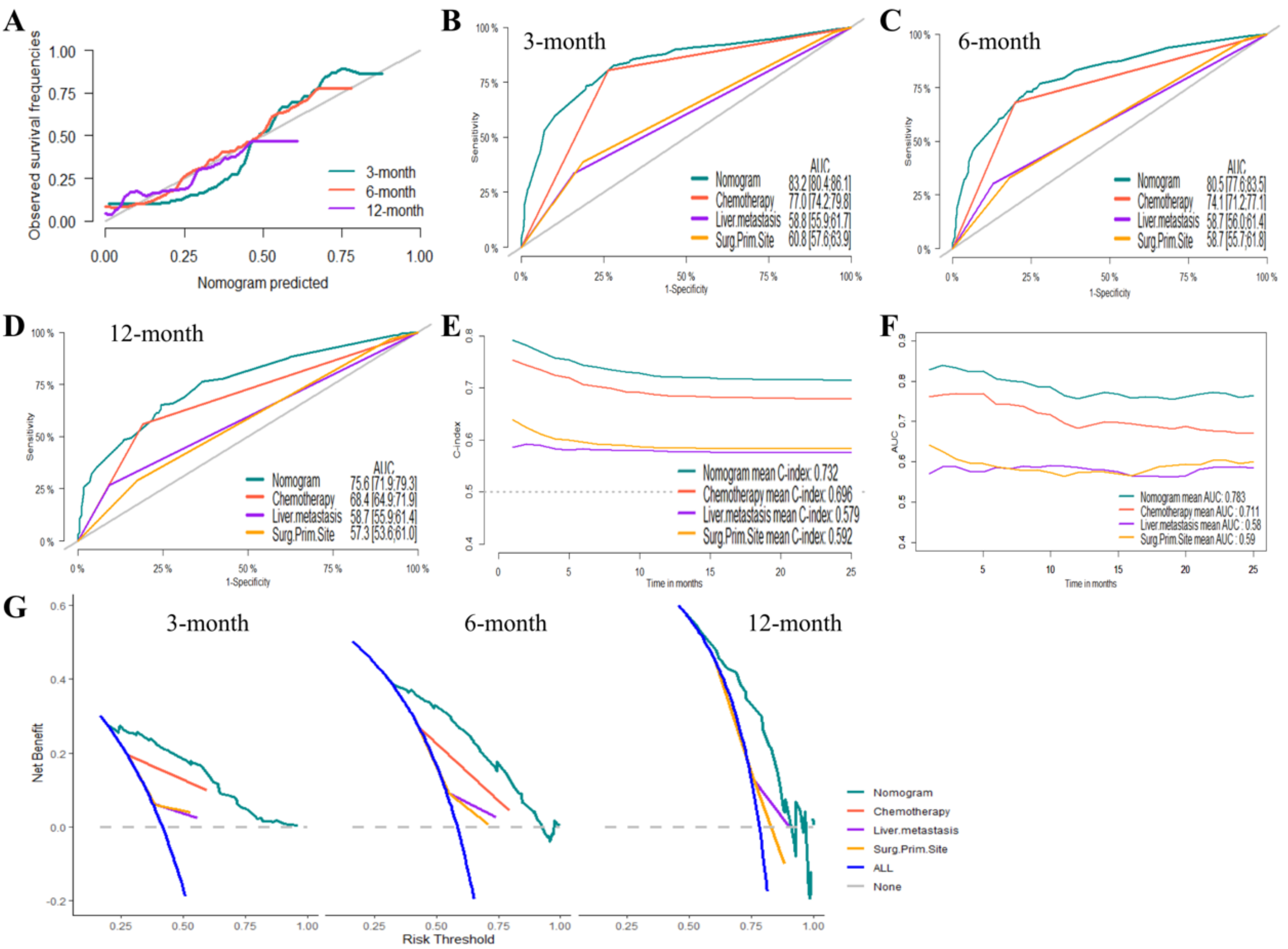

Supplementary Figure S6. Evaluation of the nomogram on training dataset for cancer-specific survival (CSS). (A) 3-, 6- and 12-month Calibration plots of Nomogram. (B) 3-month (C) 6-month and (D) 12-month Area Under the Curve (AUC) for Receiver Operating Characteristic (ROC) curves of Nomogram, Chemotherapy, Liver metastasis and Primary site surgery. (E) Overall Concordance Index (c-index) of Nomogram, Chemotherapy, Liver metastasis and Primary site surgery. (F) Overall AUC of Nomogram, Chemotherapy, Liver metastasis and Primary site surgery. (G) 3-, 6- and 12-month Decision Curve Analysis (DCA) of Nomogram, Chemotherapy, Liver metastasis and Primary site surgery.

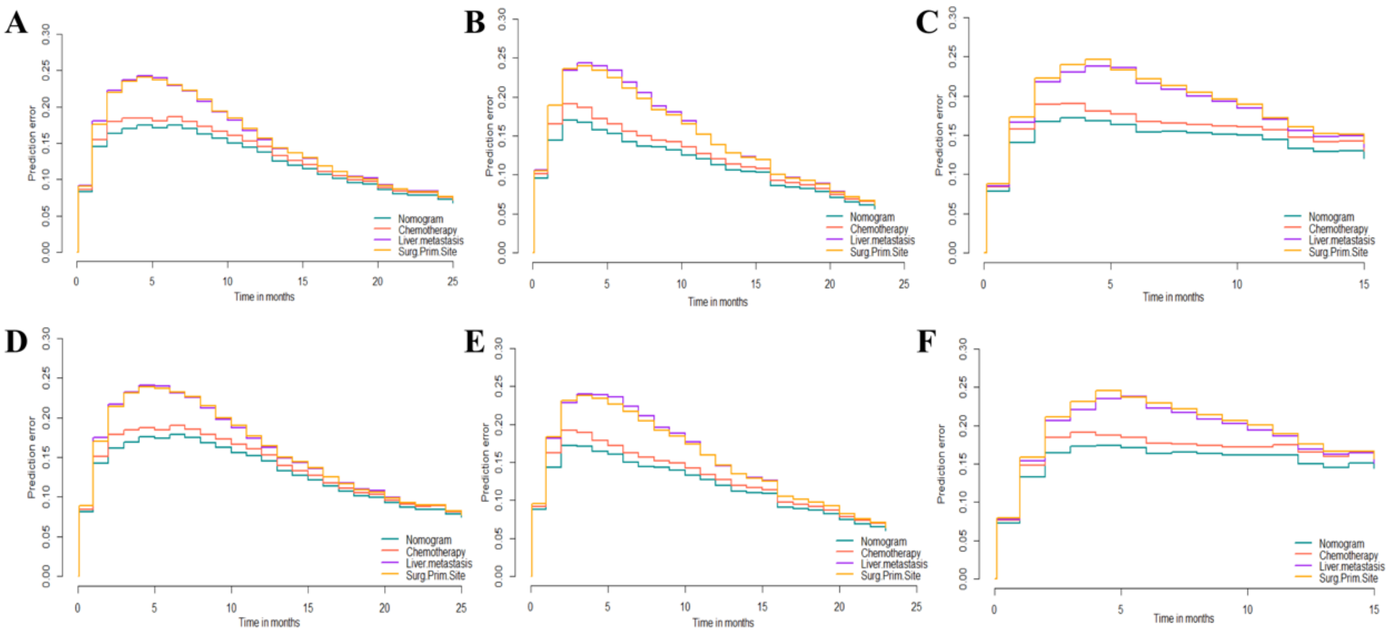

Supplementary Figure S7. The prediction error curves for different models based on the integrated Brier score (IBS). (A) Training dataset of overall survival (OS). (B) Internal validation dataset of overall survival (OS). (C) External testing dataset of overall survival (OS). (D) Training dataset of cancer-specific survival (CSS). (E) Internal validation dataset of cancer-specific survival (CSS). (F) External testing dataset of cancer-specific survival (CSS).

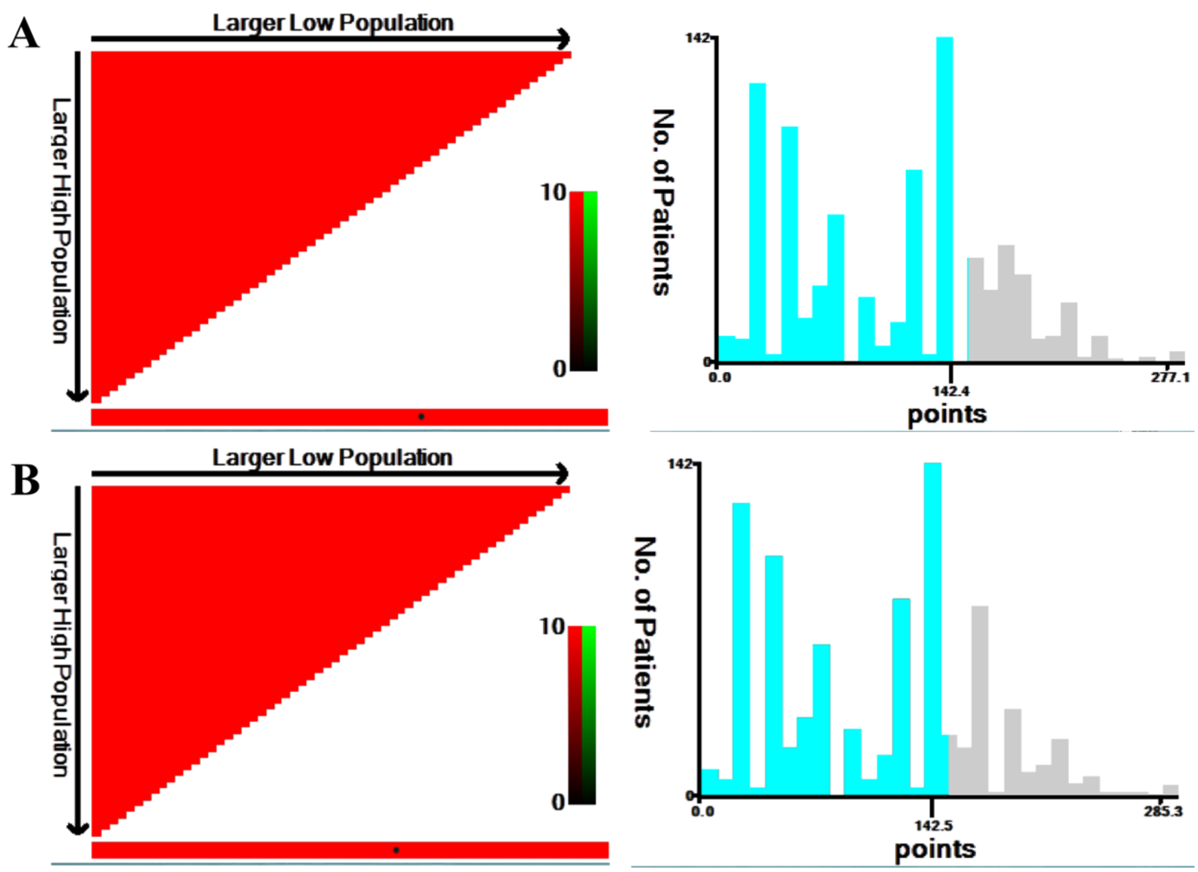

Supplementary Figure S8. Cut-off values of nomogram total points calculated by X-tile. (A) Training dataset of Overall survival (OS). (B) Training dataset of cancer-specific survival (CSS).

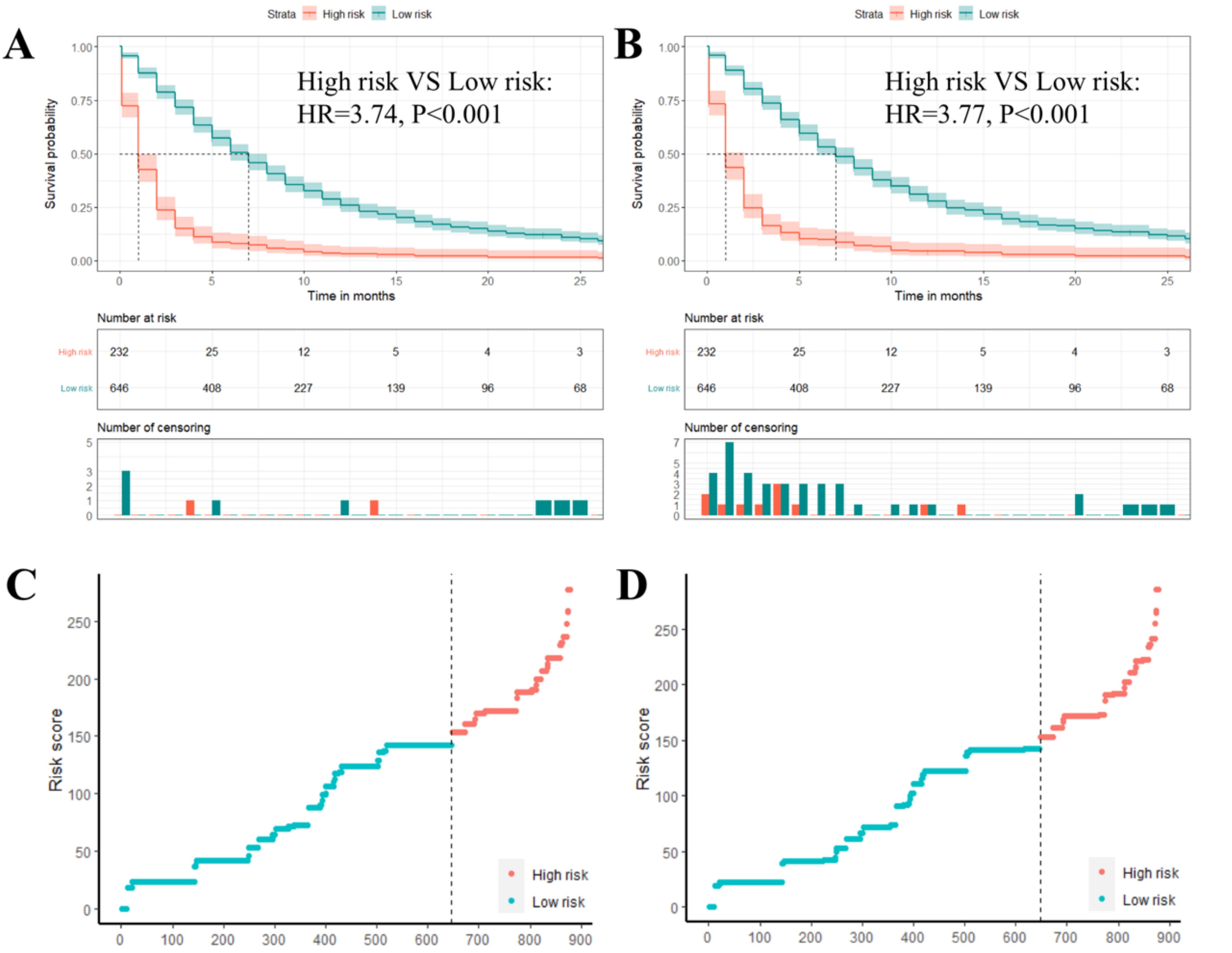

Supplementary Figure S9. Kaplan-Meier survival curves of patients stratified by risk score and scatterplot of the risk score for (A, C) Training dataset of overall survival (OS). (B, D) Training dataset of cancer-specific survival (CSS).

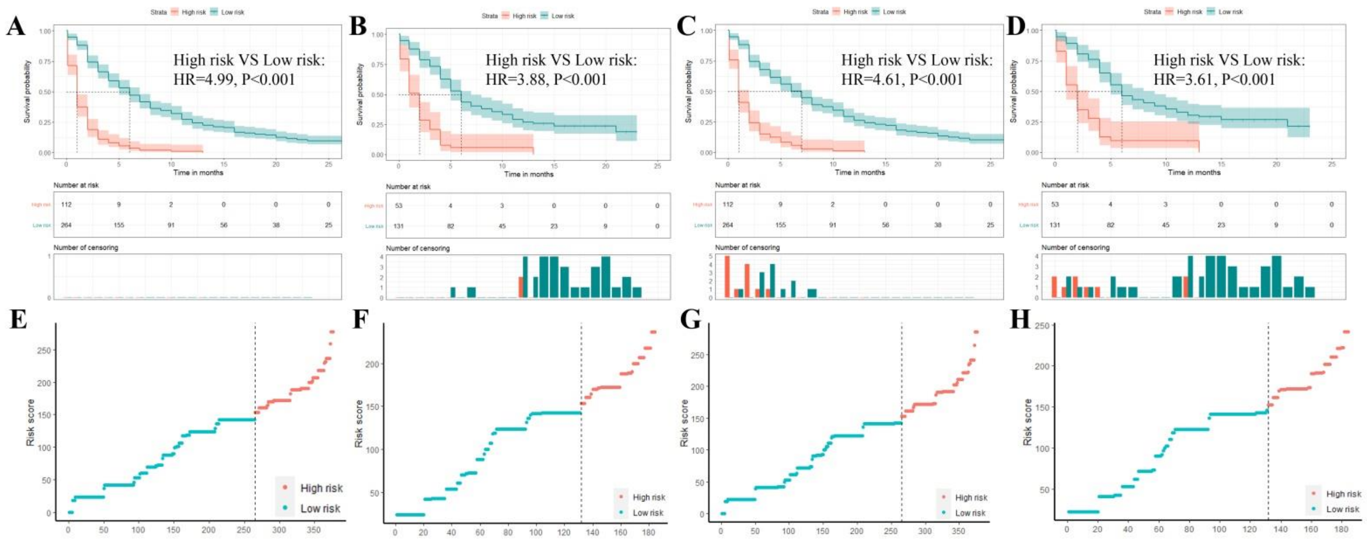

Supplementary Figure S10. Kaplan-Meier survival curves of patients stratified by risk score and scatterplot of the risk score for (A, E) Internal validation of overall survival (OS). (B, F) External testing dataset of overall survival (OS). (C, G) Internal validation dataset of cancer-specific survival (CSS). (D, H) External testing dataset of cancer-specific survival (CSS).

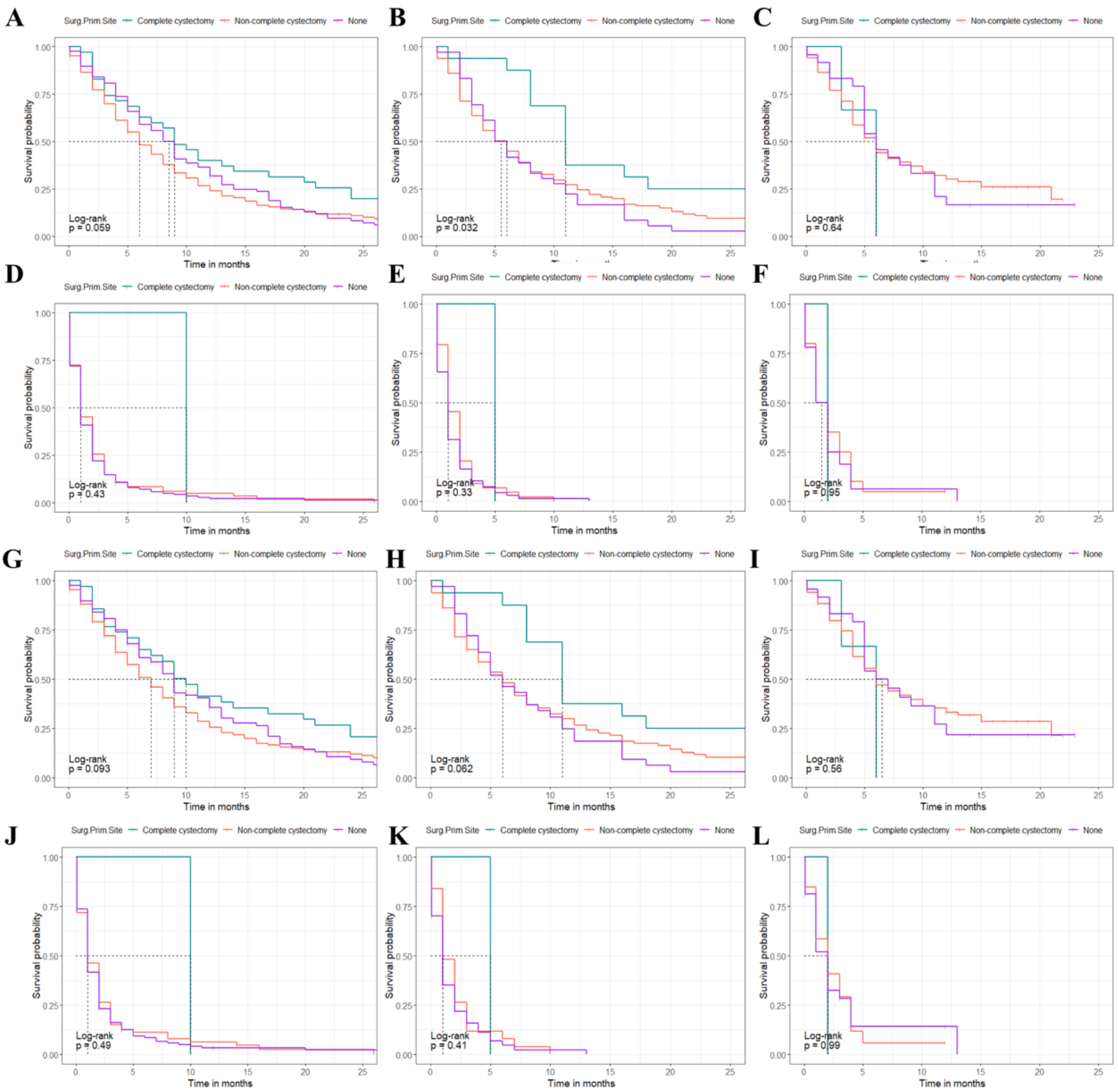

Supplementary Figure S11. Kaplan–Meier curves of patients with different surgery in low- (A-C) and high-risk (D-F) group for Overall survival (OS) and low- (G-I) and high-risk (J-L) group for Cancer-specific survival (CSS).

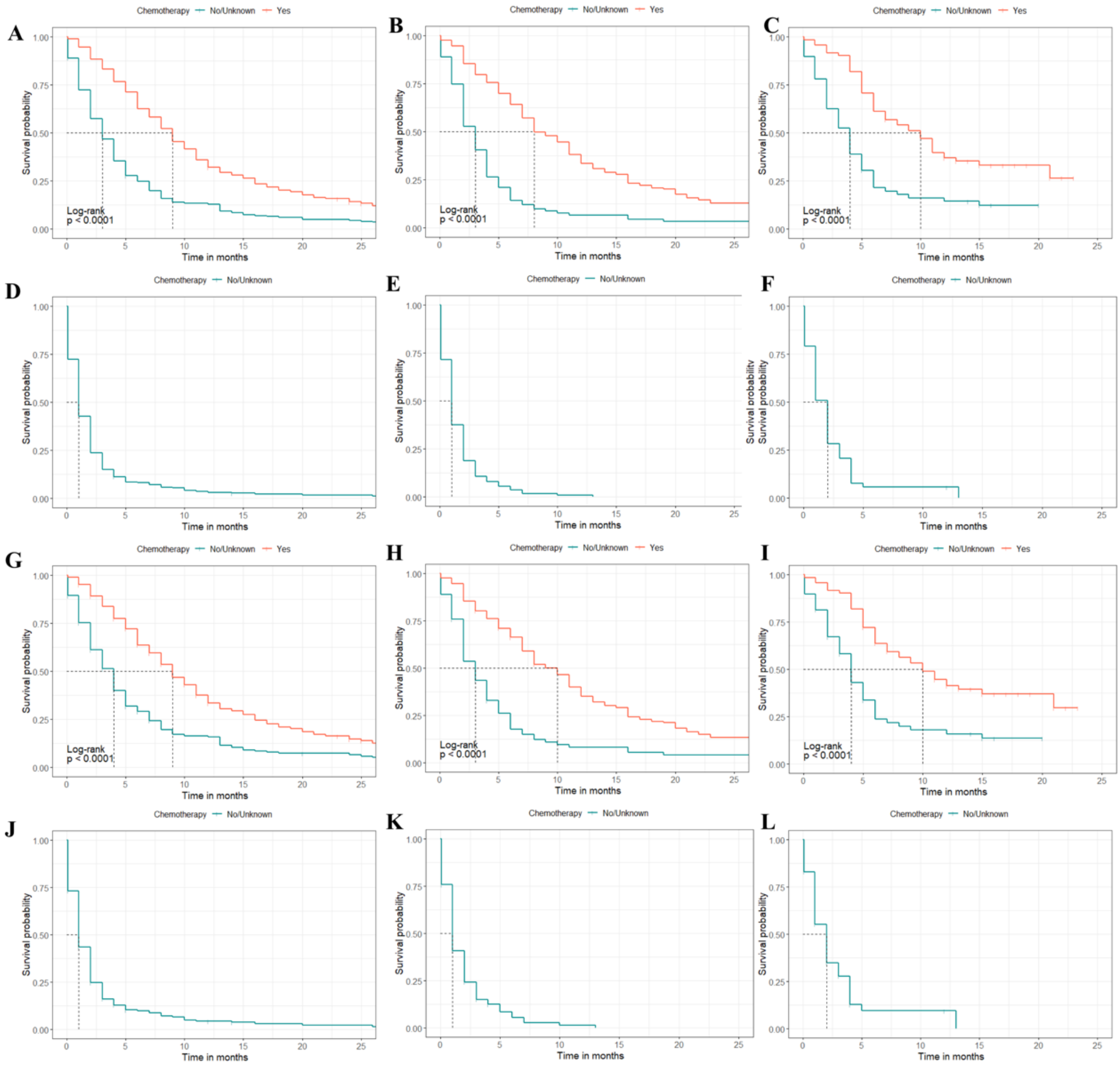

Supplementary Figure S12. Kaplan–Meier curves of patients with different chemotherapy in low- (A-C) and high-risk (D-F) group for Overall survival (OS) and low- (G-I) and high-risk (J-L) group for Cancer-specific survival (CSS).

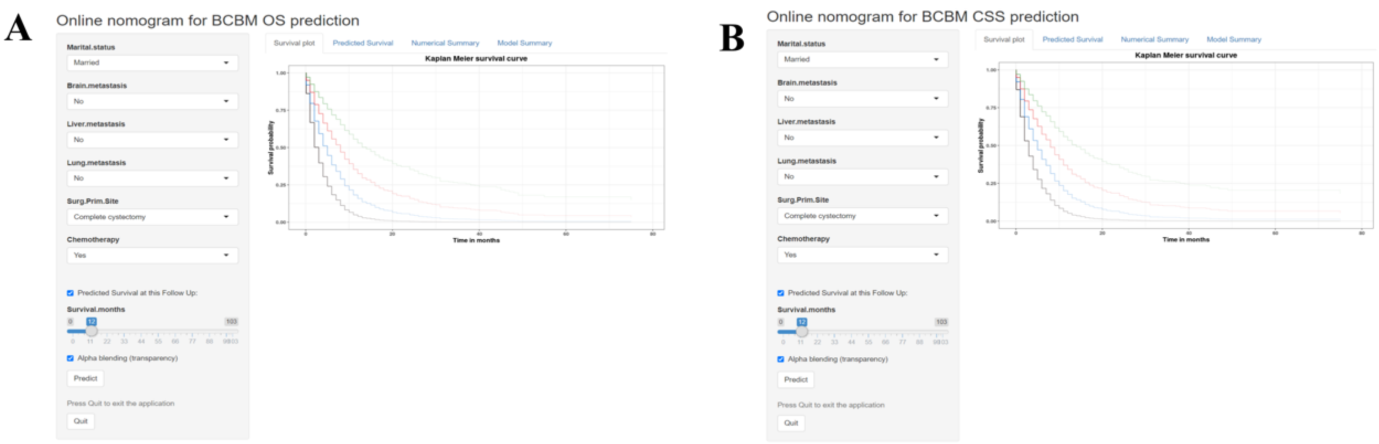

Supplementary Figure S13. The web survival rate calculator for (A) Overall survival (OS). (B) Cancer-specific survival (CSS).

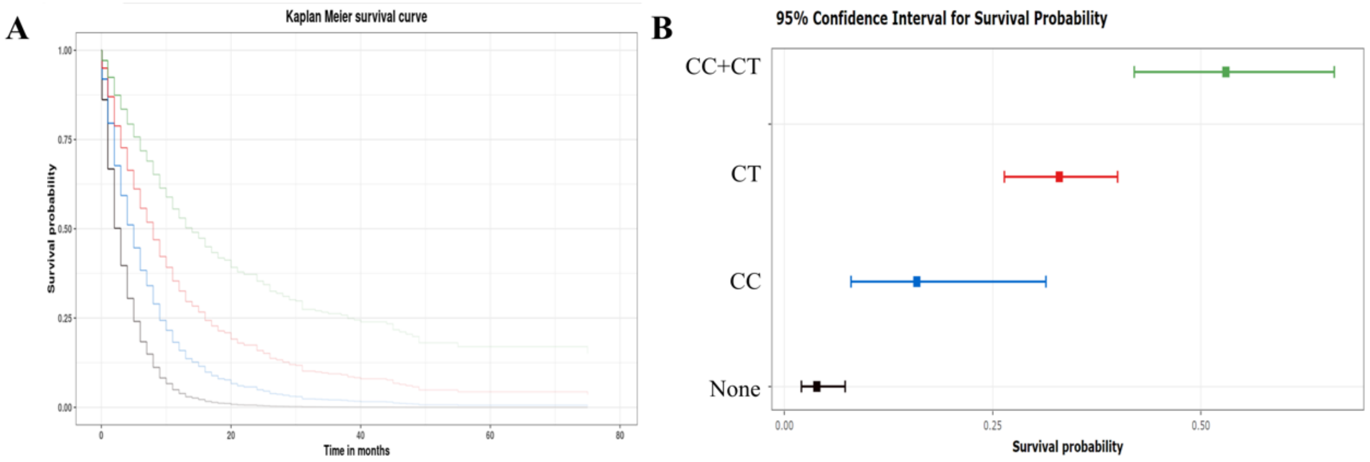

Supplementary Figure S14. The web survival rate calculator estimated overall survival (OS) of a hypothetical patient (Married; No brain metastasis, liver or lung metastasis) based on different treatment strategy. (A) Survival curves of different treatments. (B) 12-month survival probability with 95%CI of different treatments. Abbreviations: CC, complete cystectomy; CT, chemotherapy.

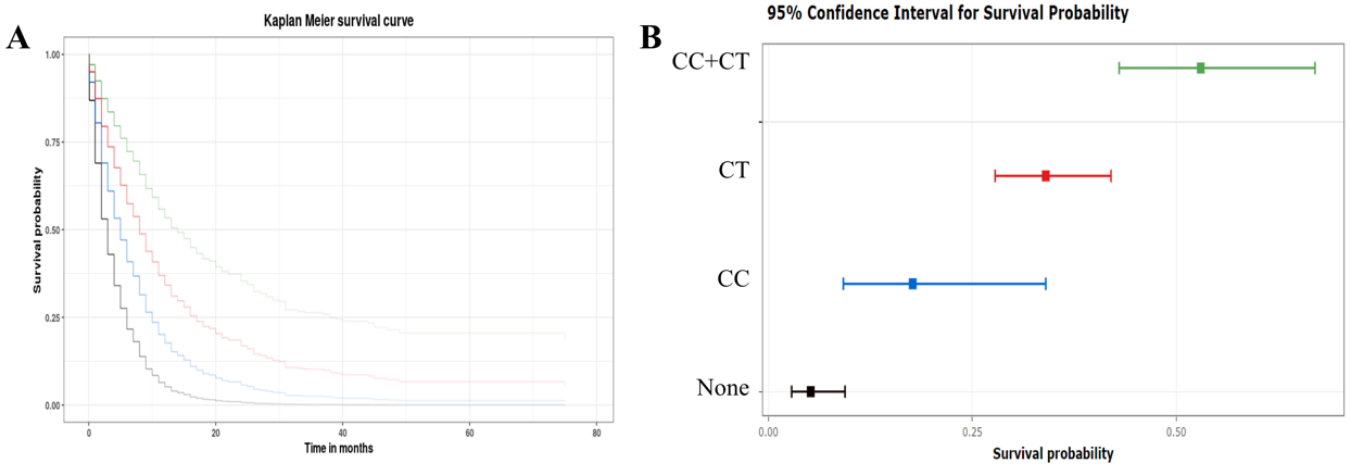

Supplementary Figure S15. The web survival rate calculator estimated cancer-specific survival (CSS) of a hypothetical patient (Married; No brain metastasis, liver or lung metastasis) based on different treatment strategy. (A) Survival curves of different treatments. (B) 12-month survival probability with 95%CI of different treatments. Abbreviations: CC, complete cystectomy; CT, chemotherapy.

Supplementary material B

STROBE Statement—Checklist of items that should be included in reports of ***cohort studies***

	Item No	Recommendation	Section and paragraph #
**Title and abstract**	1	(*a*) Indicate the study's design with a commonly used term in the title or the abstract	Page 1, Title
		(*b*) Provide in the abstract an informative and balanced summary of what was done and what was found	Page 3, Abstract
**Introduction**			
Background/rationale	2	Explain the scientific background and rationale for the investigation being reported	Page 5, Introduction, paragraph #1-#3
Objectives	3	State specific objectives, including any prespecified hypotheses	Page 5, Introduction, paragraph #3
**Materials and methods**			
Study design	4	Present key elements of study design early in the paper	Page 6-8, Methods/Section "Sources of database"; Methods/ Section "Construction and validation of the nomograms"
Setting	5	Describe the setting, locations, and relevant dates, including periods of recruitment, exposure, follow-up, and data collection	Page 6, Methods/ Section "Inclusion and exclusion criteria";
Participants	6	(*a*) Give the eligibility criteria, and the sources and methods of selection of participants. Describe methods of follow-up	Page 6, Methods/Section "Sources of database"; Methods/ Section "Inclusion and exclusion criteria"
		(*b*) For matched studies, give matching criteria and number of exposed and unexposed	Not applicable
Variables	7	Clearly define all outcomes, exposures, predictors, potential confounders, and effect modifiers. Give diagnostic criteria, if applicable	Page 6, Methods/ Section "Inclusion and exclusion criteria"
Data sources/ measurement	8*	For each variable of interest, give sources of data and details of methods of assessment (measurement). Describe comparability of assessment methods if there is more than one group	Page 6, Methods/Section "Source of database"; Methods/ Section "Inclusion and exclusion criteria"
Bias	9	Describe any efforts to address potential sources of bias	Page 6, 8, Methods/ Section "Inclusion and exclusion criteria"; Methods/ Section "Statistical analysis"
Study size	10	Explain how the study size was arrived at	Page 6, Methods/ Section "Inclusion and exclusion criteria"
Quantitative variables	11	Explain how quantitative variables were handled in the analyses. If applicable, describe which groupings were chosen and why	Page 8, Methods/ Section "Statistical analysis"
Statistical methods	12	(*a*) Describe all statistical methods, including those used to control for confounding	Page 8, Methods/ Section "Statistical analysis"
		(*b*) Describe any methods used to examine subgroups and interactions	Page 7-8, Methods/ Section "Construction and validation of the

			nomograms"; Methods/ Section "Statistical analysis"
		(*c*) Explain how missing data were addressed	Page 8, Methods/ Section "Statistical analysis"
		(*d*) If applicable, explain how loss to follow-up was addressed	Not applicable
		(*e*) Describe any sensitivity analyses	Not applicable
**Results**			
Participants	13*	(a) Report numbers of individuals at each stage of study—eg numbers potentially eligible, examined for eligibility, confirmed eligible, included in the study, completing follow-up, and analysed	Page 8-10, Results/ Section "Patient characteristics", paragraph #1; Table 1
		(b) Give reasons for non-participation at each stage	Page 8-10, Results/ Section "Patient characteristics", paragraph #1; Table 1
		(c) Consider use of a flow diagram	Figure 1
Descriptive data	14*	(a) Give characteristics of study participants (eg demographic, clinical, social) and information on exposures and potential confounders	Page 8-10, Results/ Section "Patient characteristics", paragraph #1; Table 1;
		(b) Indicate number of participants with missing data for each variable of interest	Page 8-10, Results/ Section "Patient characteristics", paragraph #1; Supplementary Table S2; Supplementary Figure S3-4;
		(c) Summarise follow-up time (eg, average and total amount)	Page 9-10, Table 1
Outcome data	15*	Report numbers of outcome events or summary measures over time	Page 9-10, Table 1
Main results	16	(*a*) Give unadjusted estimates and, if applicable, confounder-adjusted estimates and their precision (eg, 95% confidence interval). Make clear which confounders were adjusted for and why they were included	Page 11-13, Results/ Section "Independent prognostic variables and relative importance"; Results/ Section "Construction and validation of the nomograms"; Results/ Section "Risk discrimination and online calculators"; Figure 2-7; Supplementary Figure S1-4, 5-7, 9; Supplementary Table S3-4
		(*b*) Report category boundaries when continuous variables were categorized	Supplementary Figure S8; Supplementary Table S5
		(*c*) If relevant, consider translating estimates of relative risk into absolute risk for a meaningful time period	Not applicable
Other analyses	17	Report other analyses done—eg analyses of subgroups and interactions, and sensitivity analyses	Supplementary Figure S10-11
**Discussion**			
Key results	18	Summarise key results with reference to study objectives	Page 13-15, Discussion, paragraph #1-#4
Limitations	19	Discuss limitations of the study, taking into account sources of potential bias or	Page 15-16, Discussion, paragraph #5

		imprecision. Discuss both direction and magnitude of any potential bias	
Interpretation	20	Give a cautious overall interpretation of results considering objectives, limitations, multiplicity of analyses, results from similar studies, and other relevant evidence	Page 13-16, Discussion, paragraph #1-#5
Generalisability	21	Discuss the generalisability (external validity) of the study results	Page 15-16, Discussion, paragraph #5
**Other information**			
Funding	22	Give the source of funding and the role of the funders for the present study and, if applicable, for the original study on which the present article is based	Page 17, Funding

*Give information separately for exposed and unexposed groups.

Note: An Explanation and Elaboration article discusses each checklist item and gives methodological background and published examples of transparent reporting. The STROBE checklist is best used in conjunction with this article (freely available on the Web sites of PLoS Medicine at http://www.plosmedicine.org/, Annals of Internal Medicine at http://www.annals.org/, and Epidemiology at http://www.epidem.com/). Information on the STROBE Initiative is available at http://www.strobe-statement.org.

Supplementary material C

TRIPOD Checklist: Prediction Model Development and Validation

Section/Topic	Item		Checklist	Item Page
**Title and abstract**				
Title	1	D; V	Identify the study as developing and/or validating a multivariable prediction model, the target population, and the outcome to be predicted.	Page 1, Title
Abstract	2	D; V	Provide a summary of objectives, study design, setting, participants, sample size, predictors, outcome, statistical analysis, results, and conclusions.	Page 3, Abstract
**Introduction**				
Background and objectives	3a	D; V	Explain the medical context (including whether diagnostic or prognostic) and rationale for developing or validating the multivariable prediction model, including references to existing models.	Page 5, Introduction, paragraphs 1-3
	3b	D; V	Specify the objectives, including whether the study describes the development or validation of the model or both.	Page 5, Introduction, paragraphs 3
**Methods**				
Source of data	4a	D; V	Describe the study design or source of data (e.g., randomized trial, cohort, or registry data), separately for the development and validation data sets, if applicable.	Page 6-7, Methods/ Source of databases;Methods/ Feature selection
	4b	D; V	Specify the key study dates, including start of accrual; end of accrual; and, if applicable, end of follow-up.	Page 6, Methods/ Inclusion and exclusion Criteria
Participants	5a	D; V	Specify key elements of the study setting (e.g., primary care, secondary care, general population) including number and location of centres.	Page 6, Methods/ Inclusion and exclusion Criteria
	5b	D; V	Describe eligibility criteria for participants.	Page 6, Methods/ Inclusion and exclusion Criteria
	5c	D; V	Give details of treatments received, if relevant.	Not applicable
Outcome	6a	D; V	Clearly define the outcome that is predicted by the prediction model, including how and when assessed.	Page 6, Methods/ Inclusion and exclusion Criteria
	6b	D; V	Report any actions to blind assessment of the outcome to be predicted.	Not applicable
Predictors	7a	D; V	Clearly define all predictors used in developing or validating the multivariable prediction model, including how and when they were measured.	Page 6, Methods/ Inclusion and exclusion Criteria
	7b	D; V	Report any actions to blind assessment of predictors for the outcome and other predictors.	Not applicable
Sample size	8	D; V	Explain how the study size was arrived at.	Page 6, Methods/ Inclusion and exclusion Criteria; Figure 1
Missing data	9	D; V	Describe how missing data were handled (e.g., complete-case analysis, single imputation, multiple imputation) with details of any imputation method.	Page 8, Methods/ Statistical analysis
Statistical analysis methods	10a	D	Describe how predictors were handled in the analyses.	Page 7-8, Methods/ Construction and validation of the nomograms;Methods/ Statistical analysis
	10b	D	Specify type of model, all model-building procedures (including any predictor selection), and method for internal validation.	Page 7-8, Methods/ Construction and validation of the nomograms
	10c	V	For validation, describe how the predictions were calculated.	Page 7-8, Methods/ Construction and validation of the nomograms
	10d	D; V	Specify all measures used to assess model performance and, if relevant, to compare multiple models.	Page 7-8, Methods/ Construction and validation of the nomograms

	10e	V	Describe any model updating (e.g., recalibration) arising from the validation, if done.	Page 7-8, Methods/ Construction and validation of the nomograms
Risk groups	11	D; V	Provide details on how risk groups were created, if done.	Page 7-8, Methods/ Construction and validation of the nomograms
Development vs. validation	12	V	For validation, identify any differences from the development data in setting, eligibility criteria, outcome, and predictors.	Page 7-8, Methods/ Construction and validation of the nomograms; Table 1
**Results**				
Participants	13a	D; V	Describe the flow of participants through the study, including the number of participants with and without the outcome and, if applicable, a summary of the follow-up time. A diagram may be helpful.	Page 8-10, Results/ Patient characteristics; Table 1
	13b	D; V	Describe the characteristics of the participants (basic demographics, clinical features, available predictors), including the number of participants with missing data for predictors and outcome.	Page 8-10, Results/ Patient characteristics; Table 1; Supplementary Table S1-2; Supplementary Figure S3-4
	13c	V	For validation, show a comparison with the development data of the distribution of important variables (demographics, predictors and outcome).	Page 9-10, Table 1
Model development	14a	D	Specify the number of participants and outcome events in each analysis.	Page 9-10, Table 1
	14b	D	If done, report the unadjusted association between each candidate predictor and outcome.	Page 11, Results/ Independent prognostic variables and relative importance; Supplementary Table S3-4
Model specification	15a	D	Present the full prediction model to allow predictions for individuals (i.e., all regression coefficients, and model intercept or baseline survival at a given time point).	Figure 2; Supplementary Table S3-5; Supplementary Figure S12-14
	15b	D	Explain how to the use the prediction model.	Figure2; Supplementary Table S5; Supplementary Figure S12-14
Model performance	16	D; V	Report performance measures (with CIs) for the prediction model.	Page 11-12, Results/ Construction and validation of the nomograms; Figure 3-7; Supplementary Figure S5-7, 9
Model-updating	17	V	If done, report the results from any model updating (i.e., model specification, model performance).	Page 12-13, Results/ Risk discrimination and online calculators; Supplementary Figure S12-14
**Discussion**				
Limitations	18	D; V	Discuss any limitations of the study (such as nonrepresentative sample, few events per predictor, missing data).	Page 15-16, Discussion, paragraph 5
Interpretation	19a	V	For validation, discuss the results with reference to performance in the development data, and any other validation data.	Page 15, Discussion, paragraph 4
	19b	D; V	Give an overall interpretation of the results, considering objectives, limitations, results from similar studies, and other relevant evidence.	Page 13-16, Discussion, paragraph1-5
Implications	20	D; V	Discuss the potential clinical use of the model and implications for future research.	Page 16, Conclusions
**Other information**				
Supplementary information	21	D; V	Provide information about the availability of supplementary resources, such as study protocol, Web calculator, and data sets.	Page 12-13, Results/ Risk discrimination and online calculators
Funding	22	D; V	Give the source of funding and the role of the funders for the present study.	Page 17, Funding

*Items relevant only to the development of a prediction model are denoted by D, items relating solely to a validation of a prediction model are denoted by V, and items relating to both are denoted D;V. We recommend using the TRIPOD Checklist in conjunction with the TRIPOD Explanation and Elaboration document.
